# Chemical Composition, Antioxidant and Antimicrobial Activity of *Piper carpunya* and *Simira ecuadorensis*: A Comparative Study of Four Extraction Methods

**DOI:** 10.3390/plants14162526

**Published:** 2025-08-14

**Authors:** María del Cisne Guamán-Balcázar, Diana Hualpa, Garlet Infante, Luis Luzuriaga, José Luis Riofrío, Anderli Jarro, Estefany Lopez, Verónica Salas-Gomez, Rómulo Salazar, Jorge F. Reyes, Miguel A. Meneses

**Affiliations:** 1 INDETEC Research Group, Department of Chemistry, Universidad Técnica Particular de Loja, Loja 1101608, Ecuador; mcguaman@utpl.edu.ec (M.d.C.G.-B.); dihualpa@utpl.edu.ec (D.H.); jfreyes@utpl.edu.ec (J.F.R.); 2 Carrera de Alimentos, Universidad Técnica Particular de Loja, Loja 1101608, Ecuador; 3 Facultad de Ingeniería en Mecánica y Ciencias de la Producción, Escuela Superior Politécnica del Litoral—ESPOL, Campus Gustavo Galindo, Km 30.5 Vía Perimetral, Guayaquil 090902, Ecuador; verdesal@espol.edu.ec (V.S.-G.); rvsalaza@espol.edu.ec (R.S.)

**Keywords:** extraction process, ultrasound, microwave, pressurized liquid extraction, food conservation

## Abstract

In this in vitro experimental study, we compared four extraction techniques -dynamic maceration (DME), ultrasound-assisted (UAE), microwave-assisted (MAE), and pressurized liquid extraction (PLE)- to obtain bioactive extracts from two native Ecuadorian plants, *Piper carpunya* and *Simira ecuadorensis.* The effect of extraction techniques was evaluated separately for each specie based on extraction yield, total phenolic content (TPC), antioxidant capacity (DPPH, ABTS, FRAP, and ORAC assays), antimicrobial activity, and chemical composition. All analyses were performed in triplicate and analyzed statistically (ANOVA, *p* < 0.05). UAE and MAE exhibited the highest extraction yield, while PLE provided extracts with the greatest TPC. However, UAE extracts, particularly for *S. ecuadorensis*, exhibited superior antioxidant capacity across assays. GC/MS analysis revealed alkanes as predominant constituents, along with minor phenolic and ester compounds. Antimicrobial activity was observed in both species, especially against *Listeria monocytogenes* and *Pseudomonas aeruginosa*, with UAE and MAE extracts being most effective. Compounds such as isoelemicin, phytol, and ethyl linolenate may contribute to the observed bioactivities. These findings highlight the potential of *P. carpunya* and *S. ecuadorensis* as natural sources of antioxidants and antimicrobials for food and pharmaceutical applications.

## 1. Introduction

The study of new sources of natural preservatives with antioxidant and antimicrobial properties has gained significant interest, as they represent safer and more effective alternatives for delaying food spoilage, particularly in meat, fruits, and vegetables. In this context, bioactive compounds and secondary metabolites of plant origin are emerging as promising options in natural preservation [1,2,3,4,5].

Various studies have evaluated the efficacy of essential oils, such as those from *Citrus limon* var. pompia, *Cymbopogon citratus*, and *Mentha piperita* [6], as well as cinnamon bark [7], *Satureja horvatii* [8] and *Cinnamomum cassia* [9], in inhibiting *Listeria monocytogenes* in cheese, Ayran (yogurt) and meat products such as chicken, pork and ham, respectively. Similarly, *Laurus nobilis* leaf essential oil has demonstrated effectiveness in inhibiting coliforms in fresh Tuscan sausages [10], while *Citrus lemon* essential oils have been effective against *Escherichia coli* O157:H7 in apple juice [11]. Additionally, natural extracts from olive leaves [12], chestnut inner shells [13], and *Simira ecuadorensis* leaves [14], have been studied for their ability to inhibit microorganisms and control specific pathogens like *Campylobacter jejuni* in raw peeled shrimp, chicken meat, and chicken broth.

The use of medicinal plants as a primary healthcare resource is a long-standing tradition in Ecuador, a country recognized for its rich biodiversity [15]. Over the years, research has focused on identifying bioactive compounds and mechanisms underlying their health benefits [16]. While several medicinal plant species have been studied for their antioxidant and antimicrobial properties, the potential of many remains unexplored, particularly in the context of food applications. This knowledge gap motivates the evaluation of underutilized species, such as *Piper carpunya* and *Simira ecuadorensis*, for their potential as natural preservatives or functional ingredients in the food industry.

*Piper carpunya* Ruiz & Pav. (syn *Piper lenticellosum* C.D.C.), commonly known as “guaviduca”, belongs to the Piperaceae family and is native to Ecuador and Peru. It is an aromatic shrub distributed from 0 to 2000 m a.s.l. across Andean, Amazonian, and coastal regions of Ecuador [17,18]. Traditionally, it is used as an analgesic for pain management, wound treatment, and toothache relief [19]. In tropical and subtropical regions of South America, it is also employed to treat conditions including bronchitis, fever, vaginal *Candida* infections, ulcers, diarrhea, intestinal parasitic infections, and general digestive issues [17,19,20]. Chemically, the ethanolic extract of *P. carpunya* has demonstrated anti-inflammatory, antisecretory, and antimicrobial activities attributed to compounds like flavonoids, terpenoids, and phytosterols [17,21,22,23]. In methanolic extracts, Azuero et al. [24] reported antimicrobial effects against *Escherichia coli* and mild antibacterial activity against *Pseudomonas aeruginosa*. Additionally, its essential oil has shown activity against *Candida* sp. and *Klebsiella pneumoniae* [17,23].

The genus *Simira* (family Rubiaceae) comprises 45 species distributed across the Neotropics, with reported species from Mexico to Argentina [25]. *Simira ecuadorensis* is one of the most representative species in Ecuador. It grows in the dry forests of Loja (Zapotillo canton), El Oro, and Guayas provinces. In Zapotillo, it is traditionally used to wrap cheese and as wood for rural construction, where locals call it “Guápala,” while in Guayas, it is known as “Guápala roja” [26,27,28]. Its leaves are used as animal fodder and for wrapping goat milk cheeses, helping preserve them and imparting a characteristic flavor and pinkish color [27]. Recent studies have identified phenolic compounds, alkaloids, tannins, flavonoids, and terpenes in *S. ecuadorensis* [29]. Moreover, its extracts have shown antimicrobial activity against pathogens such as *Leuconostoc mesenteroides*, *Shewanella* sp., *Yersinia enterocolitica*, *Clostridium perfringens*, *Bacillus cereus*, and *Campylobacter jejuni*. Additionally, its effectiveness in inhibiting microorganisms in fish products and chicken broth has been highlighted [14].

Regarding the chemical composition, no previous studies have reported the identification of volatile compounds in natural extracts of *Piper carpunya* and *Simira ecuadorensis* However, for the essential oil of *P. carpunya*, some studies have been conducted in Ecuador. Rondón et al. [18] identified piperitone (33.97%), 1,8-cineole (11.92%), limonene (11.07%), safrole (8.18%) and α-pinene (4.49%) [18,30] in the essential oil obtained by hydrodistillation. Similarly, Ballesteros et al. [19] reported the presence of piperitone (26.2%), limonene (9.5%), elemicin (7.2%), β-phellandrene (5.6%), methyleugenol (4.5%) and 1,8-cineole (4.0%). In constrast, in *P. carpunya* from the Peruvian Amazon basin, α-terpinene (12.1%), p-cymene (10.9%), 1,8-cineole (13.0%), safrole (14.9%) and spatulenol (9.8%) were identified as major components of leaf essential oil [30]. For *Simira ecuadorensis* no information on its volatile compound profile is currently available.

Bioactive compounds derived from plant matrices hold significant potential for applications in the food, cosmetic, and pharmaceutical industries. However, their effective utilization depends on efficient extraction and separation processes [31]. Conventional methods, such as dynamic maceration extraction (DME), present several limitations, including prolonged extraction times, high energy consumption, and potential degradation of thermolabile compounds. To overcome these challenges, innovative techniques such as ultrasound-assisted extraction (UAE), microwave-assisted extraction (MAE), and pressurized liquid extraction (PLE) have gained attention due to their enhanced efficiency and selectivity [32]. From a mass transfer perspective, selecting an appropriate extraction technique is essential to overcome structural and operational resistances and to inform cost-effective scale-up strategies for industrial use.

*Piper carpunya* and *Simira ecuadorensis* were selected for this study due to their traditional use in Ecuadorian folk medicine, their incorporation in local food preparations, and the limited scientific data available on their bioactive properties. These factors highlight the need for further research to support their possible industrial applications. In this in vitro experimental study, we conducted a comparative evaluation of four widely used extraction techniques—dynamic maceration (DME), ultrasound-assisted extraction (UAE), microwave-assisted extraction (MAE), and pressurized liquid extraction (PLE)—applied separately to each specie under standardized conditions. The objective was to assess how each method influences extraction yield, total phenolic content (TPC), antioxidant capacity (DPPH, ABTS, FRAP, and ORAC assays), antimicrobial activity, and chemical composition. To our knowledge, this is the first to systematically compare these extraction techniques in these underexplored species. The findings provide valuable insights into their potential use as natural antioxidants and antimicrobials in the food industry, in alignment with the “clean label” trend, as well as in the development of topical formulations or other supplements aimed at protecting against microorganisms-induced infections.

## 2. Results

### 2.1. Yield

Table 1 presents the moisture of the plant material after the dehydration process and the extraction yields for the four extraction techniques. The moisture content was less than 10%, a value considered as a rule to avoid spoilage and for conservancy before the extraction procedures. The extraction yields ranged from 6.92% to 17.71% for *Simira ecuadorensis* and 6.88% to 17.15% for *Piper carpunya*.

For *P. carpunya*, ultrasound-assisted (UAE) and microwave-assisted extraction (MAE) achieved the highest yields (*p* < 0.05). In *S. ecuadorensis*, dynamic maceration (DME), UAE, and MAE produced relatively high yields, with no statistical differences among them. In both species, pressurized liquid extraction (PLE) consistently yielded the lowest values.

### 2.2. Total Phenols Content and Antioxidant Capacity

#### 2.2.1. Total Phenols Content

Table 2 summarizes the total phenolic content (TPC) and antioxidant capacity of the extracts obtained using each extraction method. For *Simira ecuadorensis*, TPC ranged from 22.84 to 29.99 mg GAE/100 g dm, while for *Piper carpunya* values ranged from 15.76 to 25.68 mg GAE/100 g dm. For *S. ecuadorensis*, PLE exhibited the highest TPC (*p* < 0.05), surpassing the other three extraction techniques by more than 17%, whereas no significal differences were observed among the remaining methods. For *Piper carpunya*, extracts obtained by UAE, MAE, and PLE showed similar TPC values (*p* > 0.05), all higher than those from DME.

#### 2.2.2. Antioxidant Capacity: Experimental Results

Table 2 shows significant differences in antioxidant capacity among extraction methods, as determined by DPPH, ABTS, FRAP, and ORAC assays. For *Simira ecuadorensis*, UAE provided the highest antioxidant capacity in the FRAP and ORAC assays, with values of 354.86 ± 29.37 and 625.03 ± 0.57 µmol TE/g dm, respectively. In the DPPH assay, UAE and MAE yielded the highest values (1108.68 ± 26.21 and 1025.04 ± 56.80 µmol TE/g dm, respectively). For the ABTS assay, MAE produced the highest value, while no significant difference was found between DME and UAE.

For *Piper carpunya*, DME produced the highest FRAP value (450.84 ± 4.86 µmol TE/g of dm) (*p* < 0.05). In ABTS and ORAC assays, UAE stood out with the highest antioxidant capacity values: 704.96 ± 0.84 and 525.22 ± 17.06 µmol TE/g dm, respectively. In the DPPH assay, MAE yielded the highest value (549.22 ± 32.47 µmol TE/g dm) (*p* < 0.05).

### 2.3. Antimicrobial Activity: Experimental Results

Table 3 presents the antimicrobial activity results for *P. carpunya* and *S. ecuadorensis* extracts. The extracts exhibited antimicrobial activity against four bacterial strains (*Sthapylococcus epidermidis*, *Sthapylococcus aureus*, *Listeria monocytogenes* and *Pseudomona aeruginosa*) of the eight total bacteria tested (including *Salmonella enterica* subsp., *Escherichia coli*, *Klebsiella aerogenes*, *Campylobacter jejuni*). Similarly, the extracts showed effect against *Candida albicans* with inhibition zones ranging from 8 to 15 mm and MIC between 20 and 80 mg/mL (Table 4), whereas no inhibitory effect was observed against *Aspergillus niger*.

*Listeria monocytogenes* and *Pseudomona aeruginosa* were the most sensitive bacteria to the extracts of *P. carpunya* and *S. ecuadorensis*, showing inhibition zones of 10–14 mm and 12–15 mm, respectively. No statistical differences (*p* > 0.05) were observed compared with the positive control. However, extracts obtained through PLE from both species showed no inhibition against *S. aureus* or *P. aeruginosa.* For *C. albicans*, all four extracts from *P. carpunya* exhibited lower activity (*p* < 0.05) than the positive control. Among *S. ecuadorensis* extracts DME showed inhibition which was also significantly lower (*p* < 0.05) than the positive control.

The MIC values for *P. carpunya* and *S. ecuadorensis* extracts (Table 4) ranged from 20 to 80 mg/mL, with most cases showing 80 mg/mL. For *P. carpunya*, the lowest MIC (20 mg/mL) was recorded against *L. monocytogenes* for DME, MAE and UAE extracts. MIC values of 40 mg/mL were observed for DME and MAE against *P. aeruginosa* and UAE against *S. aureus*.

For *S. ecuadorensis* the lowest MIC (20 mg/mL) was observed for MAE and UAE extracts against *P. aeruginosa*.

### 2.4. Chemical Composition of Simira ecuadoresis and Piper carpunya Extracts

Table 5 and Table 6 present the most representative volatile compounds identified and quantified for *Simira ecuadorensis* and *Piper carpunya* across the different extraction techniques. For *S. ecuadorensis*, 71 compounds were initially identified (Tables S1 and S2); however, for clarity, only the 20 most abundant or characteristic compounds are shown. The major compounds detected were: in PLE, Tetratricontane, Tetracosane, and Octacosane; in MAE, Octacosane, Tetracosane, and Docosane; in DME, Hexatriacontane, Tetracosane, and Tetratricontane; and in UAE, Ethyl palmitate, Ethyl linoleate, and Tetratricontane. In terms of compound classes, esters, alkanes and phenols predominated in *P. carpunya*, whereas *S. ecuadorensis* was mainly characterized by alkanes, esters, and siloxanes.

For *Piper carpunya*, a total of 45 volatile compounds were identified, with the most representative shown in Table 6. The main compounds by extraction technique were as follows: in PLE, isoelemicin, tetracosane and hentriacontane; in MAE, tetracosane, hentriacontane, heneicosane and hexatriacontane; in DME, hexatriacontane, hentriacontane, tetracosane and tetratriacontane; and in UAE, hexatriacontane, hentriacontane and Tetracosane. The predominant chemical groups identified were alkanes, esters, and phenols.

Supplementary Figures S1 and S2 provide the mass spectra for the most representative compounds, while Figures S3 and S4 detail the chromatographic profiles of *Piper carpunya* and *Simira ecuadorensis*, respectively.

## 3. Discussion

Extraction techniques significantly influence extraction yield. UAE and MAE were the most efficient techniques for *Simira ecuadorensis* and *Piper carpunya*. For *S. ecuadorensis*, although DME achieved yields comparable to UAE and MAE, it required two successive extraction steps, resulting in higher solvent, time, and energy consumption. These differences reflect the intrinsic characteristics of each extraction technique, the operational conditions applied, and the specific affinity of bioactive compounds in the studied species toward each process. According to Santos et al. [33], emerging extraction techniques frequently outperform traditional methods in terms of efficiency. The superior performance of UAE and MAE can be explained by their underlying mechanisms. UAE relies on ultrasound waves (>20 kHz) to generate cavitation, producing localized high temperatures and pressures that disrupt plant cell structures, thereby enhancing mass transfer of intracellular bioactive compounds into the solvent phase. Compared to conventional techniques such as Soxhlet or maceration, UAE operates at lower temperatures, reduces extraction time significantly, and improves compound recovery while using less solvent, making it a more sustainable option [33]. On the other hand, Gomez et al. [34] highlight that MAE uses electromagnetic irradiation (300 MHz to 300 GHz) to induce dipole rotation in polar solvent and solid matrices, generating heat within the sample. This rapid internal heating leads to cell wall rupture, enhancing the release of bioactive compounds. This technique ensures high extraction yields, shorter processing times, and reduces solvent use; however, its application must be carefully controlled, as high temperatures may degrade thermally sensitive compounds.

From an industrial perspective, the performance of UAE, MAE, PLE, and DME can be evaluated in terms of energy efficiency, cost, and scalability. UAE stands out for its low energy consumption and short processing time, which significantly reduces operational costs [33]. MAE also offers rapid extraction and minimal solvent use but requires careful thermal regulation [34]. Conversely, PLE operates under elevated pressures and temperatures, leading to higher energy input [35], while DME involves prolonged extraction periods and continuous agitation, which increases energy demands. Previous studies indicate that UAE and MAE can reduce energy consumption by 40–60% compared to conventional maceration, whereas PLE remains among the most energy-intensive techniques [36,37]. In terms of cost, UAE benefits from relatively low capital and operational expenses, MAE requires moderate investment, and PLE entails specialized, high-cost equipment and technical maintenance. Although DME has low initial costs, its reduced efficiency and throughput limit its competitiveness for high-value industrial applications [38]. Regarding scalability, UAE is highly adaptable to continuous industrial systems capable of processing hundreds to thousands of liters [34]. MAE and PLE are scalable but require higher capital investments and complex engineering design, while DME, despite its simplicity, typically offers limited productivity and higher resource use at scale [39]. This comparative analysis supports selecting extraction strategies that balance yield, efficiency, and industrial feasibility. The following sections examine how these extraction methods influenced antioxidant capacity, total phenolic content, antimicrobial activity, and chemical composition in both species.

### 3.1. Total Phenols

From Table 2, it is essential to consider the observation made by Osorio-Tobón [40], which emphasizes the significant role of various factors associated with extraction techniques in determining the final TPC of extracts. This highlights the inherent complexity of comparing extraction techniques due to the substantial influence of the process variables and the instability of bioactive compounds present in the plant species studied.

For *Simira ecuadorensis*, PLE yielded the highest TPC among all evaluated methods, likely due to its specific combination of operational parameters. High pressure (120 bar) improves solvent penetration into the plant matrix, promoting phenolic interaction and dissolution [41], while moderate temperature (60 °C) lowers solvent viscosity and facilitates diffusion through the solid matrix.

Conversely, UAE showed lower efficiency for *S. ecuadorensis*, possibly due to reduced cavitation at 60 °C [42]. MAE may also be constrained by solvent penetration and temperature control, limiting phenolic release [43]. The combined effect of pressure and temperature in PLE appears to overcome these limitations, emphasizing the importance of controlled operational conditions for extraction efficacy [44].

For *Piper carpunya*, MAE, UAE and PLE extracts exhibited similar TPC values (*p* > 0.05), whereas DME resulted in the lowest values. In terms of operational efficiency, UAE emerges as an optimal choice for phenolic recovery, as noted by Carreira-Casais et al. [45]. These authors emphasize UAE’s ability to reduce time, solvent, and energy consumption while preserving biomolecule integrity. Sankaran et al. [46] further report that cavitation and shear forces generated during ultrasonication enhance mass transfer, making UAE particularly effective for extracting phenolics and flavonoids. The use of polar solvents, such as water, further facilitates penetration and diffusion within plant cells.

Compared to other studies, Rondón et al. [29] reported 346.28 ± 18.60 mg GAE/100 g dm in *Simira ecuadorensis* using the Soxhlet technique with ethanol, a value far exceeding this study (29.99 ± 1.48 mg GAE/100 g dm). Similarly, Jaramillo et al. [21] reported 2.8 mg GAE/g dm in *Piper carpunya* through dynamic maceration with 70% ethanol for 24 h, surpassing our PLE result (0.26 ± 0.01 mg GAE/g dm).

Similarly, TPC in *P. carpunya* (0.26 ± 0.01 mg GAE/g dm; 1.71 ± 0.04 mg GAE/g extract) is markedly lower than in other Piper species—*P. auritum* (28.30 ± 1.99 mg GAE/g dm) and *Porophyllum ruderale* (49.07 ± 2.54 mg GAE/g dm) by UAE aqueous extracts of *P. betle* (8.3 ± 0.93), *P. betleoides* (4.1 ± 0.93), and *P. wallichii* (3.3 ± 0.57 mg GAE/g dm) [47]. For *Simira* species, Ferraz et al. [48] reported 180 mg GAE/g dm in seeds of *S. gardneriana*.

### 3.2. Antioxidant Capacity

Although there is limited information on the chemical composition of the two species studied, the antioxidant capacity in *Piper carpunya* could be attributed to compounds such as polyphenols, flavonoids, triterpenes, alkaloids, tannins, lactones, and anthraquinones [49], while in *Simira ecuadorensis*, compounds like alkaloids, phenolic acids, flavonoids, tannins, coumarins, and quinones are involved [14,29].

Antioxidant performance varied by assay and extraction technique. In *P. carpunya*, UAE achieved the highest values in ABTS and ORAC assays (*p* < 0.05), MAE in DPPH, and DME in FRAP.

For *Simira ecuadorensis*, UAE was most effective for FRAP and ORAC. For DPPH, UAE and MAE did not differ significantly (*p* > 0.05) and reported the highest values, while ABTS showed no significant differences among UAE, MAE, and DME (*p* > 0.05).

In general, after comparing the extraction techniques (DME, MAE, UAE and PLE), UAE and MAE gave the best results for each species. These results align with previous studies, which have emphasized the effectiveness of MAE and UAE in preserving and enhancing the antioxidant activity of phenolic compounds [50,51,52,53], and more broadly, that emerging extraction techniques have advantages over conventional techniques. The increased efficiency demonstrated by these techniques could be attributed to improved penetration and release of antioxidant compounds from the cellular matrices of the leaves, allowing for a more complete and effective extraction. The choice of solvent and specific operational conditions, such as temperature and treatment duration, could influence the ability of extraction methods to preserve antioxidant activity [54].

When considering industrial scaling, ultrasound technology has been applied in various food processing operations, including the extraction of bioactive compounds from plant matrices. Companies such as Weber, Hielscher, Branson, Vibracell, and REUS have developed ultrasonic extraction equipment on an industrial scale, incorporating probe and bath systems and strategically positioning transducers and reactors with capacities ranging from 30 to 1000 L, along with agitation systems to optimize the process [55]. In fact, Alzorqi et al. [56] developed a scaled model to enhance the extraction of polysaccharides from *Ganoderma lucidum* at volumes of 3 L and 6 L, starting from 0.25 L, using an ultrasonic extractor with agitation (600 W). On the other hand, Pingret et al. [57] improved the polyphenolic extraction from apple pomace at a large scale (30 L) using an ultrasonic method with 200 W tanks at 25 kHz and 40 °C for 40 min, achieving a yield 15% higher than the conventional method. Likewise, MAE has proven effective in extracting various compounds from natural products, such as antioxidants, colorants, and bio-phenols, in a rapid and reproducible manner. Microwave reactors, such as the Multiwave 3000 from Anton Paar, are used at laboratory and pilot/industrial scales. The Milestone company employs MAE technology, from the laboratory model Ethos X to the pilot/industrial MAC-75, with four 1500 W magnetrons, designed for treatments up to 75 L [55].

Antioxidant data for these species are scarce; however, in *Piper*, Luna-Fox et al. [58] reported 177 mg TE/100 g dm in *P. aduncum* (FRAP), and Luca et al. [59] reported 77 and 16.05 mg TE/g extract for *P. nigrum* and *P. longum*. These values are lower than those obtained here for *P. carpunya* (675.35 ± 14.14 mg TE/g extract; 508.19 ± 30.05 µmol TE/L). Regarding the ABTS method, Conde-Hernández et al. [60] determined values of 25.04 ± 1.63 mg TE/g dm and 50.97 ± 1.80 mg TE/g dm in ethanol-water (50:50 *v*/*v*) extracts obtained by UAE from fresh leaves of *Piper auritum* and *Porophyllum ruderale*. Additionally, da Silva et al. [61] measured antioxidant activity using the DPPH method in essential oils of *Piper aleyreanum* (412.2 ± 9.5 mg TE/mL EO), *P. anonifolium* (148.6 ± 26.9 mg TE/mL EO), *P. hispidum* (303.1 ± 49.2 mg TE/mL EO), while Ballesteros et al. [19] reported the antioxidant activity for *Piper carpunya* (71.88 ± 1.53 μmol TE/g dm). The value obtained for *Piper carpunya* is intermediate (MAE: 123.60 ± 2.16 mg/mL extract).

The leaves of *Simira ecuadorensis* are traditionally used to wrap goat cheeses to preserve them and impart flavor/color [14]. With this consideration, when comparing the FRAP results to species commonly used as condiments, such as bay leaf (504.25 ± 26.74 µmol TE/g), ginger (157.95 ± 2.2 µmol TE/g), fennel (72.4 ± 1.24 µmol TE/g), and cumin (68.7 ± 0.70 µmol TE/g) [62], it could be observed that *Simira ecuadorensis* is inferior only to bay leaf. In the case of ABTS, the results for *S. ecuadorensis* were higher than those of condiments such as ginger (39.4 ± 0.8 µmol TE/g), fennel (73.7 ± 2.8 µmol TE/g), anise (61.6 ± 0.8 µmol TE/g), cardamom (46.1 ± 2.1 µmol TE/g), and star anise (500.4 ± 14.7 µmol TE/g) [63], while at the same time it was lower than cinnamon (1119.9 ± 199.2 µmol TE/g), and clove cinnamon (2071.1 ± 75.5 µmol TE/g).

Finally, it is important to highlight that no previous studies have evaluated the antioxidant activity of *S. ecuadorensis* and *P. carpunya* using the ORAC method. The ORAC assay is a comprehensive technique that measures antioxidant capacity against biologically relevant radicals, providing information not obtained from other methods such as DPPH, ABTS, or FRAP. Notably, ORAC evaluates both the initial phase of radical neutralization and the total antioxidant capacity, including both lipophilic and hydrophilic antioxidants [64]. For comparison, Perez et al. [65] reported an ORAC antioxidant capacity of 48.3 ± 4.28 µmol TE/g DM for hexane extracts of *P. auritum* leaves. Similarly, Foffe et al. [66] reported the aqueous extracts of black *P. nigrum* (410.72 µmol TE/g DM), white *P. nigrum* (193.32 µmol TE/g DM), and *P. guineense* (340.33 µmol TE/g DM). In all cases, the ORAC values obtained in this study are higher than those previously reported in the literature.

### 3.3. Antimicrobial Activity

Extracts of *S. ecuadorensis* and *P. carpunya* exhibited significant antibacterial activity against *Listeria monocytogenes*, with inhibition zones statistically comparable to the positive control (*p* > 0.05). Similar effects have been reported for other *Piper* species, such as *Piper chaba*, whose extracts and essential oil exhibited a strong anti-listeria effect against five *Listeria monocytogenes* strains [67]. On the other hand, the study of Reyes et al. [14] reported no inhibitory activity of *S. ecuadorensis* extracts against *L. monocytogenes.* This discrepancy could be attributed to differences in extraction methods and the phytochemical composition of the leaves used. The bioactive compounds and secondary metabolites present in plant extracts can vary due to several factors, including climatic conditions, harvest stage and period, water stress, geographical location, among others [68,69,70].

Against *S. aureus*, *P. carpunya* DME, UAE, and PLE extracts exhibited strong antibacterial activity, with no statistical differences, while all *S. ecuadorensis* were also effective.

For *P. carpunya* DME, UAE and MAE, and for *S. ecuadorensis* UAE and MAE exhibited high inhibition zones, statistically equivalent to each other and to the positive control.

Conversely, both species showed low inhibitory effects against *S. epidermidis* and *C. albicans*.

The pronounced activity of the extracts against Gram-positive bacteria is consistent with their lack of an outer membrane, which in Gram-negative bacteria acts as a selective barrier, reducing permeability to antimicrobial agents [71,72]. The exception observed for *Pseudomonas aeruginosa* may indicate that phenolic compounds present in the extracts can overcome this barrier by interacting with cell structures and disrupting key physiological processes [14].

The antibacterial activity of *Piper carpunya* essential oil against *S. aureus*, *E. coli*, *K. pneumoniae* and *P. aeruginosa*, as well as its antifungal effect against *C. albicans*, has been reported previously, and this activity may be related to the presence of piperitone, the main component of oil [18,24]. Other Piper species have shown antimicrobial activity against different bacteria (Gram-positive and Gram-negative) and fungi, which are related to the presence of monoterpenes such as: 1,8-cineole, α-pinene and β-pinene [30,73]; alkylbenzenes as safrole [74] that are also present in the essential oil of *P. carpunya* [68].

Regarding *S. ecuadorensis*, it has been reported that its ethanolic and aqueous extracts exhibit antibacterial effects against *Campylobacter jejuni*, *Shewanella putrefaciens*, *Bacillus cereus*, *Yersinia enterocolitica*, *Clostridium perfringens*, *Leuconostoc mesenteroides* [14] and *Vibrio parahaemolyticus* [29]. However, unlike the present investigation, these extracts did not exhibit activity against *S. aureus*, *Pseudomonas putida*, and *Pseudomonas fluorescens*.

In studies carried out on other species of the *Simira* genus, it was found that the methanolic extracts of *Simira glaziovii* and *Simira sampaioana* against anthracnose, caused by the fungus *Colletotrichum lindemuthianum* in beans, were considered inactive [75] and that the activity against *Mycobacterium tuberculosis* and *Mycobacterium kansasii*, showed minimum inhibitory concentrations (MIC) greater than 100 μg/mL [76]. The antibacterial and antifungal activity against *S. ecuadorensis* could be attributed to the presence of phenolic compounds (phenolic acid, flavonoids, tannins, coumarins, quinones) anthraquinones [29] or alkaloids present [14,25]. To our knowledge, this is the first report of antibacterial activity of *S. ecuadorensis* against *S. epidermidis* and *C. albicans*.

Although these extracts demonstrated promising antimicrobial activity, their toxicological safety remains a critical concern for future applications. Ballesteros et al. [19] reported that the essential oil of *P. carpunya* leaves was non-genotoxic based on the Ames test, a finding consistent with Guerrini et al. [74] for the essential oils of *Piper aduncum* L. and *Piper obliquum* tested against *Salmonella* strains TA97a, TA98, TA100, and TA1535. However, Valarezo et al. [68] highlighted that safrole, a major component of *P. carpunya* oil, is classified as a probable human carcinogen and is subject to strict international regulations, underscoring the need for comprehensive risk assessment before industrial use. In contrast, no toxicological studies have been reported for *S. ecuadorensis* extracts, representing a significant knowledge gap that should be addressed through future research on safety and regulatory compliance.

### 3.4. Chemical Composition

This work presents the first qualitative chemical characterization of *Simira ecuadorensis* and *Piper carpunya* using GC/MS. This approach enabled detection of semi-volatile and extractable compounds in the extracts from four extraction methods, however, it has inherent limitations, as highly volatile compounds may have been lost during lyophilization, and more polar constituents were likely underrepresented. As shown in Table 5, differences were identified in the major compounds depending on the extraction technique used. For *S. ecuadorensis*, three different predominant compounds were observed: tetratriacontane in MAE and PLE (8.67 ± 0.10% and 11.36 ± 0.76%, respectively), hexatriacontane in DME (8.02 ± 0.11%), and ethyl linolenate in UAE (17.83 ± 0.74%).

For *P. carpunya* (Table 6), DME, UAE, and MAE showed no clear trend, with compounds such as hexatriacontane, hentriacontane, tetratriacontane, tetracosane, and docosane were identified in similar proportions (ranging from 5.11% to 8.53%). In contrast, PLE extract was characterized by isoelemicin as the major compound (13.34 ± 1.89%), while heneicosane (7.95 ± 0.25%) was found exclusively in the UAE extract.

In general, the main compounds of both species were primarily alkanes, except for isoelemicin, a phenolic compound, and ethyl linolenate, which belongs to the ester family. Regarding their biological activity, hexatriacontane and tetracosane have been reported to possess antioxidant and antimicrobial properties [77,78,79]. Hentriacontane, in turn, has shown potential for mitigating diabetes and neutralizing free radicals [80]; furthermore, its combination with 1-nonacosanol exhibits anthelmintic activity in sheep and goats [81]. Likewise, tetratriacontane has been associated with antimicrobial activity [82], while eicosane has been reported to have antifungal, cytotoxic, and antitumor properties [83,84].

Extraction efficiency can be assessed considering phenolic content, antioxidant capacity, antimicrobial activity, and chemical composition. For *P. carpunya*, the extracts obtained by UAE and PLE showed the highest TPC values (25.68 ± 0.79 and 25.58 ± 0.61 mg GAE/100 g dm, respectively) and stood out for their high antioxidant capacity in the ABTS and ORAC assays, particularly the UAE extract (704.96 ± 0.94 and 525.22 ± 17.06 µmol TE/g dm, respectively). This activity could be related to the presence of phenolic compounds such as isoelemicin, which was particularly abundant in the PLE extract, as well as the alkanes, whose antioxidant activity has also been reported.

In the case of *S. ecuadorensis*, although the highest phenolic content was obtained in the PLE extract (29.99 ± 1.48 mg GAE/100 g dm), the greatest antioxidant capacity was observed in the UAE extract, which stood out in the DPPH (1108.68 ± 26.21 µmol TE/g dm), FRAP (354.86 ± 29.37 µmol TE/g dm), and ORAC (625.03 ± 0.57 µmol TE/g dm) assays. This suggests that the chemical nature of the extracted compounds has a greater influence than the total phenolic content. Compounds like ethyl linoleate [85], ethyl linolenate [86], phytol [87], myristicin [88,89], and rosifoliol [90] in UAE may explain these results.

Regarding antimicrobial activity, most extracts from both species showed inhibition against microorganisms such as *Staphylococcus epidermidis*, *Pseudomonas aeruginosa*, *Staphylococcus aureus*, *Listeria monocytogenes*, and *Candida albicans*. The most sensitive bacteria were *L. monocytogenes* and *P. aeruginosa*, especially to the MAE and UAE extracts, which showed wide inhibition zones and low minimum inhibitory concentrations (20 mg/mL). The identified compounds may not fully account for the observed antimicrobial and antioxidant activities, which are expected to result from synergistic interactions among multiple classes of metabolites. Nevertheless, the antimicrobial activity could be influenced by the presence of bioactive compounds such as isoelemicin, phytol, nonacosane, tetratriacontane, squalene, and methyl dehydroabietate, which are present in various essential oils with known antimicrobial properties [87,91,92,93,94]. Supplementary Tables S3 and S4 summarize previous studies used for comparison and contextualize the novelty of the results presented in this work.

### 3.5. Study Limitations and Future Directions

This study was performed at a laboratory scale, which limits its direct extrapolation to industrial processes. Scaling up UAE and MAE will require addressing critical factors such as process efficiency, energy consumption, solvent recovery, and the retention of bioactivity. Pilot-scale systems typically demand higher specific power than laboratory setups, influencing both energy requirements and equipment design. Therefore, future research should include process optimization and techno-economic assessments to ensure industrial feasibility. Notably, the ethanol–water system employed in this study is Generally Recognized as Safe (GRAS), which enhances its potential for food and nutraceutical applications.

Further investigations should also incorporate advanced analytical techniques, such as LC-MS/MS and bioassay-guided fractionation, to obtain a more comprehensive chemical profile and identify compounds responsible for the observed bioactivity.

In addition, this work controlled the geographical origin and harvesting conditions of plant material to specifically isolate the effects of extraction methods. However, environmental variables such as location, climate, and season have a substantial impact on phytochemical composition and bioactivity. Therefore, future studies should include sampling across multiple sites and harvesting periods to evaluate this variability. Addressing these factors is essential for developing standardized extracts and supporting the transition to industrial-scale production.

Finally, complementary studies on extract toxicity and stability, as well as evaluations in pharmaceutical and food models, are necessary to ensure safety and efficacy prior to large-scale application.

## 4. Materials and Methods

### 4.1. Materials

Hydrochloric acid, acetic acid, iron (III) chloride hexahydrate (FeCl_3_·6H_2_O), Folin–Ciocalteu reagent, sodium bicarbonate, 1,1-Diphenyl-2-picrylhydrazyl (DPPH), gallic acid, 2,2′-Azino-bis (3- ethylbenthiazoline-6-sulfonic acid) (ABTS), potassium persulfate, 2,4,6-Tri-(2-pyridyl)-5- triazine (TPTZ), 2,2-Azobis(2-methyl-propionamidine) dihydrochloride (AAPH, 97%), (±)-6-Hydroxy-2,5,7,8-tetramethylchromane-2-carboxylic acid (Trolox, 97%), fluorescein sodium salt (C_20_H_10_Na_2_O_5_), dichloromethane, sodium sulfate anhydrous, methanol and ethanol were purchased from Sigma–Aldrich (St. Louis, MO, USA). Disodium hydrogen phosphate dodecahydrate (Na_2_HPO_4_·12H_2_O) was purchased from Merck (Darmstadt, Germany), and sodium phosphate dihydrate (NaH_2_PO_4_·2H_2_O) from Lobachemie (Mumbai, India). Medium Tripticase soy agar (TSB), Mueller hinton agar (MHA), Nutrient agar, and Brain heart broth (BHI)(TM MEDIUM), as well as Broth for molds and yeast (CYM), were purchased from DIFCO^TM^ (Sparks, NV, USA). The standard of aliphatic hydrocarbons was purchased from CHEM SERVICE (West Chester, PA, USA). Helium was purchased from INDURA (Guayaquil, Ecuador).

### 4.2. Plant Material

The guaviduca (*Piper carpunya*) leaves were collected in the Celén neighborhood, located in the rural parish of Gualel, Loja province. The guapala (*Simira ecuadorensis*) leaves were collected in Zapotillo canton, Los Limones parish, Cabeza de Toro sector, also in Loja province. Both species were harvested in February 2023 and transported to the Food Laboratory of UTPL within 4 and 8 h, respectively.

The plant samples were disinfected with calcium hypochlorite (80 ppm) and dehydrated in a tray dryer (Lassele DY-110H, Ansan, Republic of Korea) at 60 °C for 13 h. The dried leaves were reduced in size through two milling steps: first using a disc mill, followed by an ultracentrifugal mill (Retsch ZM 200, Haan, Germany) equipped with a 0.5 mm mesh. The milled material was sieved, and the fraction with particle size smaller than 250 µm was used for the extraction processes. Additionally, the final moisture content of both species was measured.

### 4.3. Extraction Process

Four extraction techniques were employed to recover bioactive compounds from *Piper carpunya* and *Simira ecuadorensis*: dynamic maceration extraction (DME), ultrasound-assisted extraction (UAE), microwave-assisted extraction (MAE), and pressurized liquid extraction (PLE). Each technique was applied independently to both plant species under standardized conditions to enable valid comparisons. The solvent system and sample-to-solvent ratio were selected based on preliminary optimization studies performed in our laboratory (at undergraduate level), aimed to maximize extraction yield and antioxidant activity. The optimal conditions identified consisted of an ethanol-water mixture (50:50 *v*/*v*) and a sample-to-solvent ratio of 1:20 (*w*/*v*). These parameters were applied consistently across all extraction techniques and plant species in this study.

#### 4.3.1. Dynamic Maceration Extraction (DME)

The mixture (sample to solvent ratio, 1:20 *w*/*v*) was heated to 45 °C in a water bath and magnetically stirred for 6 h. After this period, the liquid extract was separated by vacuum filtration. The remaining solid residue was then subject to a second extraction using the same solvent mixture, maintaining a temperature of 45 °C for an additional 3 h. Both liquid extracts were then combined and stored at 4 °C in amber glass containers to prevent potential degradation from light exposure.

#### 4.3.2. Ultrasound-Assisted Extraction (UAE)

A glass bottle containing the plant material and solvent mixture was subjected to ultrasonic treatment using an ultrasonic bath (Ultrasonic Cleaner, FS20, Fisher Scientific, Waltham, MA, USA) operating at 100 W and a frequency of 42 kHz. The extraction process was carried out for 30 min at a temperature of 60 °C. After extraction, the mixture was filtered under vacuum, and the extract was stored under refrigeration for subsequent analysis.

#### 4.3.3. Microwave-Assisted Extraction (MAE)

Microwave-assisted extraction was performed using a microwave system (Mars 6 One Touch, CEM Corporation, Matthews, NC, USA). The plant material and solvent were placed in appropriate containers, and extraction was carried out for 60 min at 1200 W and 100 °C. After completion, the extract was filtered and stored at 4 °C for subsequent analysis.

#### 4.3.4. Pressurized Liquid Extraction (PLE)

PLE was carried out using a high-pressure extraction system composed of an extraction vessel (0.25 L), a separation vessel (0.25 L), and an isocratic high-pressure pump (Knauer, Pump 40P, Berlin, Germany). Temperature was controlled by an electrical oven, while pressure regulation was maintained through a control valve [95]. The plant material was placed inside a filter paper cartridge and introduced into the extraction vessel. The vessel was then filled with the solvent, and static extraction was performed at 120 bar and 60 °C for 12 h. After this period, a continuous solvent flow (1 mL/min) was applied until reaching a total volume of 400 mL, maintaining a solid to solvent ratio of 1:20. The extract was subsequently stored at 4 °C for further analysis.

### 4.4. Chemical Composition Analysis

The obtained extracts for *S. ecuadorensis* and *P. carpunya* were dried by vacuum rotary evaporation and lyophilization and subsequently dissolved in dichloromethane to a final concentration of 4716 ppm, ensuring maximum solubilization of particles. Prior to analysis, the samples were filtered through a 0.45 µm membrane filter.

Qualitative analysis of the extracts was performed using a gas chromatograph (Agilent model 6890 N series, Agilent Technologies, Santa Clara, CA, USA) coupled to a quadrupole mass spectrometer (Agilent 5973 series inert, Agilent Technologies, Santa Clara, CA, USA).

GC/MS analyses employed a non-polar capillary column (Agilent J&W DB-5ms Ultra Inert GC column, stationary phase 5%-phenyl-methylpolyxilosane, 30 m, 0.25 mm, 0.25 µm) and an automatic injector (Agilent 7683 automatic liquid sampler, Agilent Technologies, Santa Clara, CA, USA).

The parameters used in the injection were: (a) Injector: split mode 50:1, temperature 280 °C, helium as carrier gas at 1 mL/min (constant flow), and average linear velocity 34 cm/s. (b) Oven program: initial temperature 100 °C for 3 min, ramp to 280 °C at 3 °C/min, and final isotherm for 3 min. (c) MS conditions: electron impact ionization 70 eV, electron multiplier 1600 eV, mass range 40–350 m/z, and scan rate 2 scans/s.

Compound identification was based on peak integration using Chromeleon 7 software. Retention indices (RI) were calculated following Equation (1) [96] using the retention times of standard n-alkanes (C9 to C25) analyzed under the same conditions. RI values and mass spectra were compared with reference data reported by Adams, R. [97].

(1) $$RI=100C+100\times \frac{\left({RT}_{X}-{RT}_{n}\right)}{RTN-{RT}_{n}}\;$$

where, *C*: number of carbon atoms of the aliphatic hydrocarbons (C9 to C25) eluting before the identification chemical compound; *RT_X_*: retention time of the identified chemical compound; *RT_n_*: retention time of the aliphatic hydrocarbons eluting before the identification chemical compound; and *RTN*: retention time in the hydrocarbons eluting after the identification chemical compound.

For compounds with carbon numbers >C25, identification was performed by comparing spectra with the NIST Standard Reference Data and PubChem databases, considering matches with ≥80% similarity.

GC/MS was selected as an exploratory approach for the qualitative profiling of the extracts, as no previous reports exist on the chemical composition of *Piper carpunya* and *Simira ecuadorensis*. This method enables preliminary identification of compounds through retention indices and mass spectral comparison with reference databases. While HPLC with authentic standards, provides higher specificity for phenolic and polar compounds, the lack of available standards for these species limits its applicability.

### 4.5. Determination of Total Phenols Content

The Folin-Ciocalteu procedure was applied following Swain et al. [98]. This assay is based on the colorimetric reaction between phenolic compounds and the Folin-Ciocalteu reagent at alkaline conditions, the change in color is registered by UV-Vis spectrometry at 725 nm. For analysis, 150 μL of extract were mixed with 2400 μL of distillated water and 150 μL of Folin-Ciocalteu reagent (0.25 N). The mixture was shaken for 5 min and allowed to react for 3 min before adding, 300 μL of sodium carbonate (1 N). Absorbance was then recorded, and results were expressed as mg gallic acid equivalents per gram of extract (mg GAE/g extract), using a standard curve prepared with gallic acid (0–0.5 ppm, R^2^ = 0.99).

### 4.6. Determination of Antioxidant Capacity

Antioxidant activity was assessed using DPPH, ABTS, FRAP and ORAC assays. Results were expressed as micromole of Trolox equivalent per grams of dry matter (µmol TE/g dm).

#### 4.6.1. DPPH Assay

The DPPH assay was performed according to Williams-Brand, et al. [99]. This method evaluates the ability of antioxidants to scavenge DPPH (2,2-diphenyl-1-picrylhidracyl) free radicals, indicated by a color change from purple to yellow, measured at 515 nm [100]. A DPPH stock solution was prepared by dissolving 24 mg in 100 mL of methanol; 10 mL of this solution were diluted to 50 mL and adjusted to an absorbance of 1.1 ± 0.02. For the reaction, 2850 μL of DPPH solution were mixed with 150 μL of extract and allowed to react for 24 h before measuring absorbance. Antioxidant capacity was evaluated using a Trolox standard curve (0 to 600 μMol/L).

#### 4.6.2. ABTS Assay

The procedure reported by Arnao, et al. [101] was followed for ABTS assay. An ABTS stock solution was prepared by mixing equal volumes of ABTS (7.4 mM) and potassium persulphate (2.6 mM), which reacted for 12 h in the dark at room temperature. The working solution was obtained by dissolving 1 mL of stock with 60 mL of methanol and adjusting absorbance to 1.1 ± 0.02 at 735 nm. For the assay, 2850 μL of ABTS were mixed with 150 μL of extract and incubated for 2 h before measuring absorbance at 734 nm. Results were calculated from a Trolox standard curve (to 600 μMol/L).

#### 4.6.3. FRAP Assay

FRAP assay was determined according to Benzie and Strain [102,103], with modifications from [100]. The assay measures the reduction of the ferric-TPTZ complex to its ferrous form, producing a blue color with maximum absorbance at 593 nm. The FRAP solution was obtained by mixing 25 mL of buffer acetate (pH 3.6), 2.5 mL of TPTZ (10 mM), and 25 mL of distillated water. For analysis, 2850 μL of FRAP solution were mixed with 150 μL of extract and allowed to react in the dark for 30 min. Results were calculated from a Trolox standard curve (0–600 µmol/L).

#### 4.6.4. Oxygen Radical Absorbance Capacity (ORAC) Assay

The antioxidant activity was assessed using the ORAC method adapted from García-Ruiz et al. [104]. Extracts were dissolved in methanol/HCl and diluted (1:200, 1:100, 1:50 and 1:20). Analysis was performed on a multi-modal microplate spectrophotometer (Synergy™ HTX, Santa Clara, CA, USA) controlled by the Gene 5™ version 1.1 software. Briefly, 150 µL of fluorescein solution (4 × 10^−3^ nM) and 25 µL of sample were dispensed into a black 96-well plate. Trolox standards (0–240 µM) and blanks (phosphate buffer, 75 mM, pH 7.4) were included. After incubation at 37 °C, 25 µL of AAPH solution (153 mM) were automatically added to initiate oxidation. Fluorescence readings were taken every 2 min for 2.5 h at excitation/emission wavelengths of 485/528 nm. Results were expressed as µmol TE/g dm.

### 4.7. Determination of Antimicrobial Activity

#### 4.7.1. Microbial Strains

The antimicrobial activity of the plant extracts was evaluated against ten microorganisms: eight bacteria, one mold, and one yeast. The strains were obtained from the American Type Culture Collection (ATCC) (MEDIBAC-INC S.A.S, Guayaquil, Ecuador) and are listed in Table 7.

The selection of these microorganisms was based on the report by Davydova et al. [105], which reviewed 62 studies on foodborne diseases published between 2010 and 2023. The review identified 34 commonly studied pathogens across 26 countries, highlighting frequently isolated species such as *Salmonella*, *Campylobacter*, *Escherichia coli*, *Staphylococcus aureus*, *Listeria monocytogenes*, and *Pseudomonas* spp. To ensure consistency with the experimental design, all extracts obtained from each extraction method and plant species were tested against the full panel of microorganisms to comparatively assess their antimicrobial efficacy.

#### 4.7.2. Antimicrobial Activity Assay

Antimicrobial activity was evaluated using a modified agar diffusion method [14,106], commonly used for screening plant extracts with potential food and pharmaceutical applications. Extracts were diluted to a concentration of 80 mg/mL in a 1:1 mixture of deionized water and dimethyl sulfoxide (DMSO). The microbial strains were cultured to a final density of approximately 10^8^ CFU/mL. Then, 250 µL of each microbial suspension were evenly spread onto solid agar plates. After medium solidification, wells of 8 mm diameter were aseptically punched into the agar, and 40 μL of each extract were introduced into the wells. The plates were incubated for 24 to 48 h under appropriate conditions for each microorganism, as detailed in Table 7. Antimicrobial activity was expressed as the diameter (in mm) of the inhibition zones surrounding each well. Water-DMSO solution was used as negative control, while sodium hypochlorite (0.94%) served as positive control.

For microorganisms that showed inhibition zones in the initial assay, the minimum inhibitory concentration (MIC) was determined using the same agar diffusion method. Extracts were serially diluted in water-DMSO solution to obtain concentrations of 40, 20, 10, and 5 mg extract/mL. MIC was defined as the lowest concentration (mg/mL) of extract that produced visibly inhibition of microbial growth.

### 4.8. Statistical Analysis

Statistical analysis was conducted to assess the effect of extraction technique on extraction yield, total phenolic content, antioxidant capacity, antibacterial activity, and chemical composition. A one-way analysis of variance (ANOVA) was performed separately for each plant species, with the four extraction methods (DME, UAE, MAE, and PLE) considered as treatment groups. When significant differences were detected (*p* < 0.05), mean separation was carried out using Tukey’s post hoc test. All experiments were performed in triplicate, and results are reported as mean ± standard deviation. Statistical analyses were conducted using MINITAB 18 (Minitab LLC, State College, PA, USA) at a 95% confidence level.

## 5. Conclusions

This study demonstrates that the extraction method selection plays a critical rol in determining the bioactive properties, antioxidant potential, and chemical profiles of *Piper carpunya* and *Simira ecuadorensis*. The observed differences between species and techniques highlight the need to tailor extraction processes according to the specific plant matrix. The comparative evaluation of four extraction techniques revealed that UAE and MAE were the most effective approaches for enhancing antioxidant activity and overall bioactivity. Although extracts obtained by PLE showed the highest TPC, UAE extracts consistently demonstrated the highest antioxidant capacity across various assays, emphasizing the importance of the nature of the extracted compounds over their quantity. Additionally, several extracts exhibited significant antimicrobial activity, particularly against *Listeria monocytogenes* and *Pseudomonas aeruginosa*, with low MIC, reinforcing their potential as natural antimicrobial agents. The identification of bioactive compounds such as isoelemicin, phytol, ethyl linoleate, and various alkanes contributes to a better understanding of the chemical basis underlying these activities. This study represents the first report on the ORAC antioxidant capacity and the volatile chemical composition of both species using alternative extraction techniques such as PLE, UAE, and MAE. Collectively, these findings support the feasibility of using *P. carpunya* and *S. ecuadorensis* as promising sources of natural antioxidant and antimicrobial for food preservation and health-promoting products. Future work should prioritize the optimization of operational parameters and techno-economic assessment of scaling up UAE and MAE, while ensuring bioactivity retention and industrial applicability

## Figures and Tables

**Table 1:** Extraction yields of extracts obtained by different extraction techniques.

Species	Moisture (%)	Yield (%)			
		**DME**	**MAE**	**UAE**	**PLE**
*Piper carpunya*	7.47 ± 0.01 ^a^	9.48 ± 1.44 ^aB^	17.15 ± 0.65 ^bC^	17.11 ± 0.21 ^bC^	6.88 ± 0.54 ^cA^
*Simira ecuadorenses*	5.71 ± 0.00 ^b^	16.75 ± 0.31 ^aC^	16.49 ± 0.46 ^aC^	17.72 ± 1.84 ^aC^	6.92 ± 0.25 ^bA^

DME: Dynamic maceration extraction, MAE: Microwave assisted extraction, UAE: Ultrasound assisted extraction, PLE: Pressurized Liquid extraction. ^a–c^ Different letters within the same column indicate statistical differences; ^A–C^ Different letters in the same row indicate significant differences between extraction methods, *p* < 0.05.

**Table 2:** Total phenols content and antioxidant capacity in four extracts of *Simira ecuadorensis* and *Piper carpunya*.

Species	Extraction Method	TPC mg GAE/100 g dm	DPPH μmol TE/g dm	ABTS μmol TE/g dm)	FRAP μmol TE/g dm	ORAC µmol TE/g dm
*Piper carpunya*	DME	15.76 ± 0.71 ^B^	336.67 ± 22.50 ^C^	637.78 ± 10.57 ^B^	450.84 ± 4.86 ^A^	207.06 ± 4.18 ^B^
	MAE	23.65 ± 1.02 ^A^	549.22 ± 32.47 ^A^	519.44 ± 1.90 ^C^	234.31 ± 2.37 ^B^	245.50 ± 0.04 ^C^
	UAE	25.68 ± 0.79 ^A^	466.36 ± 18.09 ^B^	704.96 ± 0.94 ^A^	236.21 ± 3.13 ^B^	525.22 ± 17.06 ^A^
	PLE	25.58 ± 0.61 ^A^	344.91 ± 19.35 ^C^	418.57 ± 10.35 ^D^	242.65 ± 5.06 ^B^	158.32 ± 0.06 ^B^
*Simira ecuadorensis*	DME	22.84 ± 0.47 ^B^	627.35 ± 30.43 ^B^	697.71 ± 14.83 ^B^	205.69 ± 10.58 ^B^	160.49 ± 0.18 ^D^
	MAE	24.64 ± 1.19 ^B^	1025.04 ± 56.80 ^A^	803.99 ± 17.04 ^A^	212.90 ± 18.93 ^B^	581.70 ± 58.74 ^A^
	UAE	24.39 ± 0.18 ^B^	1108.68 ± 26.21 ^A^	745.01 ± 24.13 ^B^	354.86 ± 29.37 ^A^	625.03 ± 0.57 ^B^
	PLE	29.99 ± 1.48 ^A^	200.98 ± 11.88 ^C^	598.27 ± 22.06 ^C^	212.33 ± 5.26 ^B^	244.14 ± 13.58 ^C^

TPC: Total phenol content, DME: Dynamic maceration extraction, MAE: Microwave assisted extraction, UAE: Ultrasound assisted extraction, PLE: Pressurized Liquid extraction. ^A–D^ Different letters within the same column indicate statistical differences, *p* < 0.05.

**Table 3:** Antimicrobial activity of extracts of *P. carpunya* and *S. ecuadorensis* expressed as the diameter of the inhibition halo (mm).

	Microorganism	Extraction Technique				Positive Control
		DME	MAE	UAE	PLE	
*P. carpunya*	Gram-positive					
	*L. monocytogenes*	12.7 ± 2.1 ^A^	14.0 ± 4.6 ^A^	12.0 ± 3.5 ^A^	11.3 ± 0.6 ^A^	16.3 ± 1.2 ^A^
	*S. aureus*	9.7 ± 1.2 ^BC^	8.7 ± 0.6 ^C^	10.7 ± 0.6 ^B^	10.0 ± 1.7 ^BC^	17.3 ± 0.6 ^A^
	*S. epidermidis*	8.3 ± 0.6 ^B^	8.0 ± 0.0 ^B^	9.0 ± 1.7 ^B^	ni	19.3 ± 0.6 ^A^
	Gram-negative					
	*S. enterica* subsp. *enterica*	ni	ni	ni	ni	16.7 ± 1.5
	*E. coli* *P. aeuruginosa*	ni 10.3 ± 2.1 ^A^	ni 13.3 ± 1.5 ^A^	ni 12.0 ± 2.7 ^A^	ni ni	18.0 ± 0.0 14.0 ± 2.0 ^A^
	*C. jejuni*	ni	ni	ni	ni	10.0 ± 1.0
	*K. aerogenes*	ni	ni	ni	ni	19.7 ± 0.6
	Fungi					
	*A. niger*	ni	ni	ni	ni	31.0 ± 1.7
	*C. albicans*	8.3 ± 0.6 ^BC^	9.0 ± 0.0 ^B^	8.7 ± 0.6 ^BC^	8.0 ± 0.0 ^C^	28.3 ± 0.6 ^A^
*S. ecuadorensis*	Gram-positive					
	*S. epidermidis* *S. aureus * *L. monocytogenes*	8.3 ± 0.6 ^B^ 8.7 ± 0.6 ^B^ 15.0 ± 4.0 ^A^	8.0 ± 0.0 ^B^ 8.3 ± 0.6 ^B^ 12.7 ± 2.1 ^A^	8.0 ± 0.0 ^B^ 8.3 ± 0.6 ^B^ 12.0 ± 3.6 ^A^	ni 9.3 ± 1.5 ^B^ 12.0 ± 1.5 ^A^	20.7 ± 2.1 ^A^ 18.0± 1.0 ^A^ 16.7± 1.5 ^A^
	Gram-negative					
	*S. enterica* subsp. *enterica* *E. coli*	ni ni	ni ni	ni ni	ni ni	16.7 ± 1.5 18.0 ± 0.0
	*K. aerogenes*	ni	ni	ni	ni	19.7 ± 0.6
	*P. aeuruginosa*	ni	12.7 ± 2.1 ^A^	12.3 ± 1.5 ^A^	ni	14.7 ± 1.1 ^A^
	*C. jejuni*	ni	ni	ni	ni	10.0 ± 1.0
	Fungi					
	*A. niger*	ni	ni	ni	ni	31.0 ± 1.7
	*C. albicans*	8.3 ± 0.6 ^B^	ni	ni	ni	28.3 ± 0.6 ^A^

ni, no inhibition; positive control: sodium hypochlorite solution (0.94%); results are expressed as mean ± standard deviation (*n* = 3). ^A–C^ Different letters within the same row indicate statistical differences, *p* < 0.05; DME (dynamic maceration extraction), MAE (microwave-assisted extraction), UAE (ultrasound-assisted extraction), and PLE (pressurized liquid extraction).

**Table 4:** Minimum inhibitory concentration (MIC) (mg/mL) of *Piper carpunya* and *S ecuadorensis* extracts.

Microorganism	*P. carpunya*				*S. ecuadorensis*			
	DME	MAE	UAE	PLE	DME	MAE	UAE	PLE
*Staphylococcus epidermidis*	80	80	80	_	80	80	80	_
*Pseudomona aeruginosa*	40	40	80	_	80	20	20	_
*Staphylococcus aureus*	80	80	40	80	80	80	80	80
*Listeria monocytogenes*	20	20	20	80	80	80	80	80
*Candida albicans*	80	80	80	80	80	_	_	_

Extracts concentration range 5–80 mg/mL; three replicates (*n* = 3); DME (dynamic maceration extraction), MAE (microwave-assisted extraction), UAE (ultrasound-assisted extraction), and PLE (pressurized liquid extraction).

**Table 5:** Most representative volatile compounds identified in different extracts of *Simira ecuadorensis.* of *Simira ecuadorensis*.

TR (min)	Peak Name	CAS Number	PLE	MAE	DME	UAE	IC	KI_C_	KI_L_	M+ m/z
32.14	Eicosane	112-95-8	0.18 ± 0.08 A	0.29 ± 0.02 A	2.39 ± 0.16 B	-	KI	2006	2000	
33.05	7,9-Di-tert-butyl-1-oxaspiro(4,5)deca-6,9-diene-2,8-dione	82304-66-3	2.17 ± 0.06 A	2.65 ± 0.051 BC	2.66 ± 0.16 BC	0.99 ± 0.04 D	KI	1931	1938	
35.25	Ethyl palmitate	628-97-7	2.01 ± 0.05 A	2.39 ± 0.06 A	-	6.59 ± 0.24 B	MS			57, 43, 85, 101, 239, 284
35.93	Harman	486-84-0	-	1.65 ± 0.16 A	0.47 ± 0.10 B	3.32 ± 0.05 C	MS			182, 154,77, 127, 91
40.52	Ethyl Linoleate	544-35-4	1.06 ± 0.02 A	0.74 ± 0.02 B	-	5.83 ± 0.33 C	MS			67, 81, 55, 95, 41, 109
40.74	Ethyl linolenate	1191-41-9	2.46 ± 0.04 A	1.86 ± 0.00 A	1.02 ± 0.28 A	17.83 ± 0.74 B	MS			79, 67, 93, 55, 41, 108, 121, 135
41.42	Docosane	629-97-0	3.79 ± 0.11 A	4.19 ± 0.14 A	3.59 ± 0.14 A	4.01 ± 0.14 A	KI	2206	2200	
43.40	N,N-Dimethylpalmitamide	3886-91-7	3.00 ± 0.13 A	1.33 ± 0.16 BC	3.01 ± 0.13 A	1.28 ± 0.08 BC	MS			87, 72, 100, 45
44.14	Octadecane, 3-ethyl-5-(2-ethylbutyl)-	55282-12-7	2.87 ± 0.05 A	2.51 ± 0.24 A	0.85 ± 0.26 BC	0.63 ± 0.17 BC	MS			57, 71, 43, 85, 99
46.94	Tetracosane	646-31-1	4.88 ± 0.50 A	5.87 ± 0.14 B	5.08 ± 0.10 A	-	MS			43, 57, 71, 85, 99
52.03	Octacosane	630-02-4	4.69 ± 0.13 A	6.08 ± 0.12 B	4.20 ± 0.22 A	3.45 ± 0.08 C	MS			57, 71, 43, 85, 99, 113, 127, 141
56.13	Phthalic acid, 5-methylhex-2-yl nonyl ester	-	2.39 ± 0.04 A	3.95 ± 0.08 BC	2.42 ± 2.42 BC	1.61 ± 0.47 A	MS			149, 57, 71, 43, 97, 167, 293
56.91	Squalene	111-02-4	-	3.19 ± 0.08	-	-	MS			69, 81, 95, 121, 137, 149, 207, 293
57.90	Phthalic acid, 4-methylpent-2-yl nonyl ester	-	0.50 ± 0.00 A	0.32 ± 0.10 A	-	4.04 ± 0.19 B	MS			149, 57, 71, 85, 97, 167, 127, 207, 293
58.98	Octadecane, 3-ethyl-5-(2-ethylbutyl)-	55282-12-7	1.65 ± 0.14 A	1.83 ± 0.01 A	2.10 ± 0.72 A	-	MS			57, 43, 69,97, 81, 111
61.13	Tetratriacontane	14167-59-0	8.67 ± 0.10 A	11.36 ± 0.76 B	4.32 ± 0.16 CD	4.78 ± 0.04 CD	MS			57, 71, 85, 43, 97, 111, 127, 141
62.31	2,4,6-Tris(1-phenylethyl)phenol	18254-13-2	1.56 ± 0.03 A	1.24 ± 0.13 B	2.89 ± 0.09 C	1.57 ± 0.14 A	MS			57, 69, 47, 391, 43, 83, 406, 313
69.63	γ-Sitosterol	83-47-6	0.74 ± 0.12 A	-	2.04 ± 1.43 A	-	MS			57, 69, 97, 43, 81, 111, 207, 281, 414, 329
71.62	Hexatriacontane	630-06-8	2.62 ± 0.08 A	3.51 ± 0.24 B	8.02 ± 0.11 C	-	MS			57, 71, 43, 85, 97
79.06	Methyl 3,5-dicyclohexyl-4-hydroxybenzoate	55125-23-0	-	2.68 ± 0.17 A	1.63 ± 0.05 B	0.94 ± 0.07 C	MS			57, 69, 317, 207, 191, 43
	Total identified (%)		75.09	78.95	74.97	78.79				
	Alkanes (%)		35.18	39.68	34.92	18.29				
	Esters (%)		16.63	19.45	14.53	41.8				
	Siloxanes (%)		15.72	3.66	5.31	5.75				

PLE (pressurized liquid extraction), MAE (microwave-assisted extraction), DME (dynamic maceration extraction), UAE (ultrasound-assisted extraction), IC (Identification Criteria), KI_C_ (Calculated Kovats index), KI_L_ (Literature Kovats index), M+ (The molecular ion) and MS (Mass Spectra). A–D Different letters within the same row indicate statistical differences, *p* < 0.05.

**Table 6:** Most representative volatile compounds identified in different extracts of *Piper carpunya*.

Ret. Time	Peak Name	CAS Number	DME	MAE	UAE	PLE	IC	KI_C_	IK_L_	M+ m/z
22.13	Diethyl Phthalate	84-66-2	0.89 ± 0.28 A	1.92 ± 0.27 B	0.66 ± 0.02 A	2.70 ± 0.18 C	KI	1614	1603	
24.30	Isoelemicin	5273-85-8	2.87± 1.49 A	4.49 ± 0.05 A	3.46 ± 0.26 A	13.34 ± 1.89 B	MS			193, 208, 133, 105, 165,79
28.73	Nonadecane	629-92-5	1.25 ± 0.47 A	3.36 ± 0.05 B	1.39 ± 0.11 A	0.72	MS			57, 43, 71, 85, 91
34.30	Dibutyl phthalate	84-74-2	1.30 ± 0.40 A	1.62 ± 0.38 A	0.23 ± 0.02 B	-	KI	1971	1967	
35.06	Eicosane	112-95-8	4.23 ± 0.68 A	7.45 ± 0.03 B	4.32 ± 0.09 A	-	MS			57, 43, 71, 85, 91, 99, 163
40.75	(Z,Z,Z)-9,12,15-Octadecatrienoic acid, ethyl ester	1191-41-9	2.59 ± 1.70 A	-	1.30 ± 0.02 A	5.68 ± 0.48 C	MS			79, 67, 95
41.05	Heneicosane	629-94-7	-	7.95 ± 0.25	-	-	MS			57, 71, 43, 85
41.41	Docosane	629-97-0	5.11 ± 1.30 A	-	5.68 ± 0.11 A	6.06 ± 0.59 A	MS			57, 71, 43, 85
41.76	Acetic acid n-octadecyl ester	822-23-1	0.92 ± 0.00 A	1.90 ± 0.02 B	0.89 ± 0.04 A	2.12 ± 0.08 C	KI	2218	2211	
43.25	N,N-Dimethylpalmitamide	3886-91-7	1.83 ± 0.16 A	2.80 ± 0.24 BC	1.45 ± 0.19 A	3.30 ± 0.25 BC	MS			87, 55, 43, 72
46.89	Tetracosane	646-31-1	6.61 ± 1.00 A	8.53 ± 0.21 BCD	7.23 ± 0.61 ADE	8.74 ± 1.03 BCE	MS			57, 71, 43, 85, 97, 113, 127, 210
52.03	Hentriacontane	630-04-6	7.04 ± 1.75 A	8.33 ± 0.38 A	7.46 ± 0.32 A	8.39 ± 0.48 A	MS			57, 43, 71, 85, 97, 111, 125, 207
54.43	Nonacosane	630-03-5	-	1.90 ± 0.97 A	1.30 ± 0.11 A	2.59 ± 0.32 A	MS			57, 71, 43, 85, 97
55.85	1,3-Benzenedicarboxylic acid, bis(2-ethylhexyl) ester	137-89-3	1.40 ± 0.26 A	3.36 ± 0.27 BC	1.55 ± 0.11 A	2.94 ± 0.47 BC	MS			149, 167, 57, 71
60.85	Hexatriacontane	630-06-8	7.99 ± 1.61 A	7.92 ± 0.00 A	8.53 ± 0.06 A	-	MS			43, 57, 71, 85, 97, 113, 127, 141, 155, 207, 281
65.54	Tetratriacontane	14167-59-0	6.47 ± 2.45 A	5.58 ± 0.76 A	7.18 ± 0.63 A	0.69 ± 0.36 A	MS			57, 71, 43, 81, 97, 111, 127, 141, 253
71.18	Tetratetracontane	7098-22-8	4.51	2.05 ± 2.63 A	-	-	MS			57, 71, 85, 43, 97, 111, 207, 125
71.65	Hexatriacontane	630-06-8	6.03	2.72 ± 0.00 A	5.27 ± 0.48 B	5.53 ± 0.55 B	MS			57, 71, 43, 85, 97, 11, 207, 281
74.10	Sesquiterpene lactone (epoxidized) *	-	1.93 ± 0.63 A	0.91 ± 0.86 A	2.05 ± 0.22 A	3.44 ± 1.02 B	MS			Tentatively identified based on MS fragmentation 385, 69, 83, 97
	Total identified (%)		73.26	85.81	71.89	83.07				
	Alkanes (%)		52.32	59.45	50.64	35.76				
	Esters (%)		12.7	13.73	8.06	20.66				
	Phenols (%)		4.8	6.31	6.6	16.78				

PLE (pressurized liquid extraction), MAE (microwave-assisted extraction), DME (dynamic maceration extraction), UAE (ultrasound-assisted extraction), IC (Identification Criteria), KI_C_ (Calculated Kovats index), KI_L_ (Literature Kovats index), M+ (The molecular ion) and MS (Mass Spectra). A–E Different letters within the same row indicate statistical differences, *p* < 0.05. * Retention indices were not determined due to the absence of alkane standards.

**Table 7:** Microorganisms evaluated for antimicrobial activity.

Microorganism	Strain	Gram *	Incubation Temperature (°C)	Broth	Test Agar
*Salmonella enterica* subsp. *enterica*	VT000312-10EA derived ATCC 14028	-	37	TSB	Muller Hinton
*Escherichia coli*	ATCC 11775	-	37	BHI	Muller Hinton
*Klebsiella aerogenes*	ATCC 13048	-	37	BHI	Muller Hinton
*Staphyloccoccus epidermidis*	ATCC 12228	+	37	BHI	Muller Hinton
*Pseudomona aeuruginosa*	ATCC 10145	-	37	TSB	Muller Hinton
*Campylobacter jejuni*	ATCC 33560	-	42	BHI	Nutrient + 5% blood
*Staphyloccoccus aureus*	ATCC 25923	+	37	BHI	Muller Hinton
*Listeria monocytogenes*	ATCC 19115	+	37	BHI	Muller Hinton
*Aspergillus niger*	ATCC 6275		25	CYM	Muller Hinton
*Candida albicans*	ATCC 24433		25	CYM	Muller Hinton

American Type Culture Collection (ATCC). * -: Gram-negative bacteria; +: Gram-positive bacteria.

## Data Availability

The original contributions presented in the study are included in the article/Supplementary Materials, further inquiries can be directed to the corresponding author.

## Supplementary Materials

The following supporting information can be downloaded at: https://www.mdpi.com/article/10.3390/plants14162526/s1, Figure S1: Mass spectra of representative volatile constituents identified in *Piper carpunya* by GC–MS; Figure S2: Mass spectra of representative volatile constituents identified in *Simira ecuadorensis* by GC–MS; Figure S3: Chromatographic profile of volatile constituents from *Piper carpunya* analyzed by GC-MS. (1) Isoelemicin, (2) (Z,Z,Z)-9,12,15-Octadecatrienoic acid, ethyl ester, (3) Heneicosane, (4) Tetracosane, (5) Hentriacontane, (6) Hexatriacontane; Figure S4: Chromatographic profile of volatile constituents from *Simira ecuadorensis* analyzed by GC-MS. (1) Ethyl palmitate, (2) Ethyl Linoleate, (3) Ethyl linolenate, (4) Docosane, (5) Octacosane, (6) Tetratriacontane; Table S1: Total volatile compounds identified in different extracts of *Simira ecuadorensis* [107,108]; Table S2: Total volatile compounds identified in different extracts of *Piper carpunya* [109,110]; Table S3: Summary of previous studies on *Simira ecuadorensis* and related species [14,25,29,111,112]; Table S4; Summary of previous studies on *Piper carpunya* and related species [18,19,24,30,68,113,114,115].
