# Supplementary material for: Chemical Composition, Antioxidant and Antimicrobial Activity of Piper carpunya and Simira ecuadorensis: A Comparative Study of Four Extraction Methods
Source: Plants (Basel). 2025 Aug 14;14(16):2526. doi: 10.3390/plants14162526 (PMC12389035; doi:10.3390/plants14162526)
Supplement: Supplementary file 1 [file plants-14-02526-s001.zip › Tables S1 and S2 _ R2.pdf]

Table S1. Total volatile compounds identified in different extracts of *Simira ecuadorensis.*

TR (min)	Peak Name	CAS Number	PLE	MAE	DME	AUE	IC	KI_C	IK_L	M+ m/z
**18.55**	Butylated hydroxytoluene	128-37-0	0.74 ± 0.02 A	-	0.93 ± 0.07 B	0.36 ± 0.00 C	KI	1519	1514	
**19.00**	Myristicin	607-91-0	0.88 ± 0.01 A	-	0.96 ± 0.04 A	0.43 ± 0.02 B	KI	1531	1517	
**21.76**	Hexadecane <n->	71963-77-4	0.23 ± 0.00 AC	0.32	0.41 ± 0.04 BD	0.54 ± 0.26 CD	KI	1606	1600	
**22.13**	Rosifoliol	84-66-2	0.68 ± 0.02 A	0.86 ± 0.02 BD	0.79 ± 0.10 AD	0.29 ± 0.00 C	KI	1616	1600	
**22.74**	d-Selinene	473-14-3	0.32 ±0.02	-	-	-	KI	1633	1638	
**27.12**	Amorpha-4,9-diene <7,14-anhydro->	394251-67-3	0.86 ± 0.07	-	-	-	KI	1755	1755	
**28.08**	Cyclocolorenone <epi->	1911-75-7	0.27 ± 0.03	-	-	-	KI	1783	1774	
**28.87**	Octadecane	593-45-3	0.29 ± 0.01 A	0.50 ± 0.04 B	0.73 ± 0.05 CD	0.67 ± 0.03 CD	KI	1805	1800	
**29.35**	Cryptomeridiol	4666-84-6	0.32 ± 0.00	-	-	-	KI	1820	1813	
**29.17**	Cyclopentadecanolide	106-02-5	-	-	-	0.56 ± 0.01	KI	1814	1832	
**29.87**	Isopropyl tetradecanoate	110-27-0	0.52 ± 0.01 A	0.21 ± 0.00 B	-	-	MS			228, 43, 102, 57, 211, 73
**31.50**	Octasiloxane, 1,1,3,3,5,5,7,7,9,9,11,11,13,13,15,15-hexadecamethyl-	19095-24-0	-	-	-	0.34 ± 0.24	MS*			73, 207, 147, 221, 281, 43, 57
**32.14**	Eicosane	112-95-8	0.18 ± 0.08 A	0.29 ± 0.02 A	2.39 ± 0.16 B	-	KI	2006	2000	
**33.05**	7,9-Di-tert-butyl-1-oxaspiro(4,5)deca-6,9-diene-2,8-dione	82304-66-3	2.17 ± 0.06 A	2.65 ± 0.051 BC	2.66 ± 0.16 BC	0.99 ± 0.04 D	KI	1931	1938	
**34.36**	Dibutyl phthalate	84-74-2	-	-	0.29 ± 0.03 A	0.20 ± 0.01 B	KI	1982	1975	
**34.66**	Dibutyl phthalate	84-74-2	0.27 ± 0.05 A	0.09	0.23 ± 0.01 B	-	KI	1982	1975	
**34.94**	1,4a-Dimethyl-7-(propan-2-yl)-1,2,3,4,4a,9,10,10a-octahydrophenanthrene	5323-56-8	-	-	0.29 ± 0.02	-	MS			241, 159, 213, 43, 256
**35.25**	Ethyl palmitate	628-97-7	2.01 ± 0.05 A	2.39 ± 0.06 A	-	6.59 ± 0.24 B	MS			57, 43, 85, 101, 239, 284
**35.93**	Harman	486-84-0	-	1.65 ± 0.16 A	0.47 ± 0.10 B	3.32 ± 0.05 C	MS			182, 154,77, 127, 91
**36.36**	Juvabione	17904-27-7	0.74 ± 0.08 A	-	0.76 ± 0.17 A	-	KI	2037	2018	

**36.52**	Kaurene	34424-57-2	0.25 ± 0.01 A	0.32	0.41 ± 0.12 A	0.34 ± 0.06 A	KI	2042	2042	
**38.17**	Heneicosane <n->	629-94-7	1.4	-	1.11 ± 0.04 A	0.87 ± 0.14 A	KI	2106	2100	
**38.41**	1-Decanol, 2-hexyl-	2425-77-6	1.74 ± 0.00 A	1.92 ± 0.03 B	0.38 ± 0.05 CD	0.27 ± 0.05 CD	MS			57, 43, 71, 85, 99, 113, 127
**39.02**	Phytol	150-86-7	0.54 ± 0.02 A	0.27 ± 0.06 B	-	0.99 ± 0.01 C	MS			71, 57,43, 81, 123, 95, 111
**40.52**	Ethyl Linoleate	544-35-4	1.06 ± 0.02 A	0.74 ± 0.02 B	-	5.83 ± 0.33 C	MS			67, 81, 55, 95, 41, 109
**40.74**	Ethyl linolenate	1191-41-9	2.46 ± 0.04 A	1.86 ± 0.00 A	1.02 ± 0.28 A	17.83 ± 0.74 B	MS			79, 67, 93, 55, 41, 108, 121, 135
**41.42**	Docosane	629-97-0	3.79 ± 0.11 A	4.19 ± 0.14 A	3.59 ± 0.14 A	4.01 ± 0.14 A	KI	2206	2200	
**41.57**	Hexadecanamide	629-54-9	0.25 ± 0.01 A	0.59 ± 0.01 B	0.64 ± 0.09 C		MS			59, 43, 72, 83, 97, 111
**41.91**	Octadecyl acetate	822-23-1	1.60 ± 0.04 A	0.38 ± 0.10 B	1.43 ± 0.15 A	1.21 ± 0.07 A	MS			43, 83, 97, 55, 69, 111, 61
**42.43**	(S)-1-(hydroxymethyl)ethane-1,2-diyl dipalmitate	761-35-3	-	-	-	0.34 ± 0.01	MS			57, 43, 69, 81, 95
**42.66**	Incensole oxide	21698-66-8	0.25 ± 0.05 A	-	0.26	0.25 ± 0.02 A	KI	2250	2280	
**43.05**	Jatrorrhizine	3621-38-3	0.70 ± 0.07	0.27	0.26		MS			57, 282, 43, 69, 83, 97, 338, 173, 323, 267
**43.40**	N,N-Dimethylpalmitamide	3886-91-7	3.00 ± 0.13 A	1.33 ± 0.16 BC	3.01 ± 0.13 A	1.28 ± 0.08 BC	MS			87, 72, 100, 45
**43.62**	Cyclononasiloxane, octadecamethyl-	556-71-8	0.68 ± 0.04 A	0.71 ± 0.15 A	0.70 ± 0.05 A	1.14 ± 0.46 B	MS			87, 57, 43, 73, 95, 147, 221, 281, 355, 429
**43.95**	Tricosane	638-67-5	-	-	0.35 ± 0.02 A	1.55 ± 0.08 B	MS			57, 43, 71, 85, 95, 109, 125, 137
**43.98**	1-Phenanthrenecarboxaldehyde, 1,2,3,4,4a,9,10,10a-octahydro-1,4a-dimethyl-7-(1-methylethyl)-, [1R-(1α,4aβ,10aα)]-	13601-88-2	-	0.59 ± 0.05	-	-	MS			57, 43, 71, 173, 209, 243, 269, 284, 241
**44.14**	Octadecane, 3-ethyl-5-(2-ethylbutyl)-	55282-12-7	2.87 ± 0.05 A	2.51 ± 0.24 A	0.85 ± 0.26 BC	0.63 ± 0.17 BC	MS			57, 71, 43, 85, 99
**45.15**	Hentriacontane	630-04-6	0.20 ± 0.08 A	0.27 ± 0.05 A	0.29	0.22 ± 0.07	MS			57, 71,43, 85, 97, 111
**45.90**	Methyl dehydroabietate	1235-74-1	0.18 ± 0.03 A	-	0.32 ± 0.00 B	-	MS			57, 43, 239, 71, 85, 97, 111

**46.94**	Tetracosane	646-31-1	4.88 ± 0.50 A	5.87 ± 0.14 B	5.08 ± 0.10 A	-	MS	43, 57, 71, 85, 99
**47.41**	17-Pentatriacontene	6971-40-0	0.93 ± 0.08 A	0.50 ± 0.03 B	1.37 ± 0.02 C	0.90 ± 0.10 A	MS	43, 57, 69, 83, 97, 111, 125, 120
**48.21**	9-Octadecenoic acid, (2-phenyl-1,3-dioxolan-4-yl)methyl ester, cis-	56599-45-2	0.29 ± 0.02 A	-	0.29 ± 0.07 A	-	MS	55, 43, 87, 69, 97, 83, 125, 238
**48.36**	Phenol, 2,4-bis(1-phenylethyl)-	2769-94-0	0.50 ± 0.00 A	-	0.76 ± 0.02 B	0.45 ± 0.08 A	MS	57, 43, 71, 287, 302, 81, 97, 115
**49.52**	Heptacosane	593-49-7	1.72 ± 0.07 A	1.06 ± 0.19 BC	1.69 ± 0.22 A	0.76 ± 0.05 BC	MS	57, 71, 43, 85, 97
**49.97**	Phenol, 2,4-bis(1-phenylethyl)-	2769-94-0	0.36 ± 0.03 A	-	0.85 ± 0.18 B	0.31 ± 0.04 C	MS	57, 287, 43, 69, 97, 83, 302, 111, 209
**50.06**	1-Chloroheptacosane	62016-79-9	0.23 ± 0.02	-	-	-	MS	57, 43, 69, 97, 81, 315, 111,
**50.22**	Cyclononasiloxane, octadecamethyl-	556-71-8	1.31 ± 0.17 A	0.59 ± 0.24 B	1.10 ± 0.52 C	0.83 ± 0.33 D	MS	73, 355, 57, 429, 147
**50.75**	Bis(2-ethylhexyl) phthalate	117-81-7	0.81 ± 0.16 A	1.47 ± 0.02 B	0.53 ± 0.02 C	0.43 ± 0.01 D	MS*	149, 57, 43, 71, 167, 279
**52.03**	Octacosane	630-02-4	4.69 ± 0.13 A	6.08 ± 0.12 B	4.20 ± 0.22 A	3.45 ± 0.08 C	MS	57, 71, 43, 85, 99, 113, 127, 141
**52.55**	Heptadecane, 9-hexyl-	55124-79-3	0.16 ± 0.01 A	0.35 ± 0.1 B	0.29 ± 0.00 C	0.18 ± 0.06 A	MS	57, 43, 69, 97, 83, 111, 123
**53.20**	Cyclononasiloxane, octadecamethyl-	556-71-8	1.11 ± 0.03 A	0.65 ± 0.00 B	1.17 ± 0.13 A	0.92 ± 0.17 C	MS	73, 57, 127, 355, 429, 221, 147
**54.43**	Tetratetracontane	7098-22-8	1.74 ± 0.05 A	1.77 ± 0.15 A	1.63 ± 0.08 B	0.90 ± 0.09 C	MS	57, 71, 43
**55.15**	Phthalic acid, nonyl 4-octyl ester	-	0.27 ± 0.09 A	0.29 ± 0.06 A	-	-	MS	57, 43, 71, 97, 83, 149, 111, 293
**55.50**	Phthalic acid, 4,4-dimethylpent-2-yl nonyl ester	-	1.76 ± 0.01A	1.71 ± 0.06 A	1.40 ± 1.40 A	1.61 ± 0.09 A	MS	149, 57, 71, 43, 85, 97, 167, 293
**55.82**	Bis(2-ethylhexyl) iso phthalate	137-89-3	1.29 ± 0.03 A	1.77 ± 0.08 A	1.20 ± 1.20 A	-	MS	149, 57, 70, 43, 83, 261, 167
**56.00**	Cyclononasiloxane, octadecamethyl-	556-71-8	0.81 ± 0.01 A	0.71 ± 0.01 A	0.82 ± 0.82 A	0.74 ± 0.51 A	MS	57, 355, 73, 221, 149, 429
**56.13**	Phthalic acid, 5-methylhex-2-yl nonyl ester	-	2.39 ± 0.04 A	3.95 ± 0.08 BC	2.42 ± 2.42 BC	1.61 ± 0.47 A	MS	149, 57, 71, 43, 97, 167, 293
**56.91**	Squalene	111-02-4	-	3.19 ± 0.08	-	-	MS	69, 81, 95, 121, 137, 149, 207, 293
**57.90**	Phthalic acid, 4-methylpent-2-yl nonyl ester	-	0.50 ± 0.00 A	0.32 ± 0.10 A	-	4.04 ± 0.19 B	MS	149, 57, 71, 85, 97, 167, 127, 207, 293

**58.59**	Cyclononasiloxane, octadecamethyl-	556-71-8	0.63 ± 0.02 A	0.41 ± 0.06 B	0.73 ± 0.25 A	0.54 ± 0.20 A	MS	149, 57, 69, 43, 97, 293
**58.88**	Phthalic acid, nonyl pentadecyl ester	-	0.61 ± 0.12 A	0.35 ± 0.22 A	0.38 ± 0.13 A	0.47 ± 0.22 A	MS	149, 57, 71, 43, 85, 97
**58.98**	Octadecane, 3-ethyl-5-(2-ethylbutyl)-	55282-12-7	1.65 ± 0.14 A	1.83 ± 0.01 A	2.10 ± 0.72 A	-	MS	57, 43, 69,97, 81, 111
**59.40**	Phthalic acid, hept-4-yl nonyl ester	-	0.79 ± 0.06 A	1.24 ± 0.04 A	1.14 ± 0.39 A	0.34 ± 0.03 B	MS	149, 57, 43, 71, 293
**60.24**	Cholestan-3-one, cyclic 1,2-ethanediyl aetal, (5ß)-	25328-53-4	0.36 ± 0.07 A	0.47 ± 0.03 B	0.41 ± 0.14 AB	-	MS	57, 69, 97, 81, 43, 109, 125
**60.43**	Decasiloxane, docosamethyl-	556-70-7	0.29 ± 0.01 A	0.59 ± 0.57 AB	0.79 ± 0.23 B	0.79 ± 0.64 AB	MS	57, 69, 97, 81, 43, 221, 207
**60.77**	Tetracosamethyl-cyclododecasiloxane	18919-94-3	-	-	-	0.45 ± 0.26	MS	57, 69, 97, 43, 111, 221, 281, 355, 429, 207
**61.13**	Tetratriacontane	14167-59-0	8.67 ± 0.10 A	11.36 ± 0.76 B	4.32 ± 0.16 CD	4.78 ± 0.04 CD	MS	57, 71, 85, 43, 97, 111, 127, 141
**62.31**	2,4,6-Tris(1-phenylethyl)phenol	18254-13-2	1.56 ± 0.03 A	1.24 ± 0.13 B	2.89 ± 0.09 C	1.57 ± 0.14 A	MS	57, 69, 47, 391, 43, 83, 406, 313
**69.63**	γ-Sitosterol	83-47-6	0.74 ± 0.12 A	-	2.04 ± 1.43 A	-	MS	57, 69, 97, 43, 81, 111, 207, 281, 414, 329
**71.62**	Hexatriacontane	630-06-8	2.62 ± 0.08 A	3.51 ± 0.24 B	8.02 ± 0.11 C	-	MS	57, 71, 43, 85, 97
**79.06**	Methyl 3,5-dicyclohexyl-4-hydroxybenzoate	55125-23-0	-	2.68 ± 0.17 A	1.63 ± 0.05 B	0.94 ± 0.07 C	MS	57, 69, 317, 207, 191, 43
	**Total identified (%)**		75.09	78.77	74.97	78.05		
	**Alkanes (%)**		35.18	39.68	34.92	18.29		
	**Esters (%)**		16.63	19.45	14.53	41.8		
	**Siloxanes (%)**		15.72	3.66	5.31	5.75		

PLE (pressurized liquid extraction), MAE (microwave-assisted extraction), DME (dynamic maceration extraction), UAE (ultrasound-assisted extraction), IC (Identification Criteria), KI (Kovats Index) and MS (Mass Spectra); MS*(compound identified based on mass spectra from the reference [107] and [108])

Table S2. Total volatile compounds identified in different extracts of *Piper carpunya.*

Ret. Time	Peak Name	CAS Number	DME	MAE	UAE	PLE	IC	KIc	IKL	M+ m/z
18.48	Benzene, 1,2-dimethoxy-4-(1-propenyl)-	93-16-3	-	0.91	1.09 ± 0.15	-	MS			178, 107, 163, 91
21.70	Cyclooctasiloxane, hexadecamethyl-	556-68-3	0.69 ± 0.09 A	-	0.72 ± 0.02 B	-	KI	1604	1693	
22.13	Diethyl Phthalate	84-66-2	0.89 ± 0.28 A	1.92 ± 0.27 B	0.66 ± 0.02 A	2.70 ± 0.18 C	KI	1614	1603	
24.30	Isoelemicin	5273-85-8	2.87± 1.49 A	4.49 ± 0.05 A	3.46 ± 0.26 A	13.34 ± 1.89 B	MS			193, 208, 133, 105, 165, 79, 91
24.96	Octadecane, 3-ethyl-5-(2-ethylbutyl)-	55282-12-7	0.25 ± 0.12 A	-	0.26 ± 0.06 A	-	MS			71, 43, 57, 85
28.73	Nonadecane	629-92-5	1.25 ± 0.47 A	3.36 ± 0.05 B	1.39 ± 0.11 A	0.72	MS			57, 43, 71, 85
29.50	Isopropyl myristate	110-27-0	0.53 ± 0.19 A	1.00 ± 0.03 B	0.55 ± 0.09 A	-	KI	1833	1823	
34.58	1,2-Benzenedicarboxylic acid, monobutyl ester	131-70-4	0.76 ± 0.70	-	-	-	MS			149
34.30	Dibutyl phthalate	84-74-2	1.30 ± 0.40 A	1.62 ± 0.38 A	0.23 ± 0.02 B	-	KI	1971	1967	
35.06	Eicosane	112-95-8	4.23 ± 0.68 A	7.45 ± 0.03 B	4.32 ± 0.09 A	-	MS			57, 43, 71, 85, 91, 99, 163
36.29	Tricyclo[4.2.1.1(2,5)]dec-3-ene-9,10-dione	-	-	-	0.63 ± 0.06	-	MS			134, 91, 79, 106
36.44	Dehydroabietine	5323-56-8	-	-	0.31 ± 0.04	-	MS			241, 159
37.26	10,18-Bisnorabieta-8,11,13-triene	32624-67-2	-	-	0.43 ± 0.00	-	MS			227, 242, 143, 55, 69
38.48	Carbonic acid, eicosyl vinyl ester	2243791-78-6	0.92 ± 0.33 A	1.61 ± 0.04 BC	1.02 ± 0.06 A	2.18 ± 0.29 BC	MS			57, 71, 43, 85, 99
39.03	Phytol	150-86-7	0.35 ± 0.02 A	-	0.32 ± 0.02 A	1.09 ± 0.06 B	KI	2125	2122	
39.48	Octadecane, 3-ethyl-5-(2-ethylbutyl)-	55282-12-7	-	-	0.18	-	MS*			44, 57, 69, 85
40.54	Linoleic acid ethyl ester	544-35-4	0.94 ± 0.35 A	-	0.64 ± 0.00 B	1.92 ± 0.03 C	MS			67, 41, 81
40.75	(Z,Z,Z)-9,12,15-Octadecatrienoic acid, ethyl ester	1191-41-9	2.59 ± 1.70 A	-	1.30 ± 0.02 B	5.68 ± 0.48 C	MS			57, 71, 43, 85
41.05	Heneicosane	629-94-7	-	7.95 ± 0.25	-	-	MS			79, 67, 95
41.41	Docosane	629-97-0	5.11 ± 1.30 A	-	5.68 ± 0.11 A	6.06 ± 0.59 A	MS			57, 71, 43, 85
41.76	Acetic acid n-octadecyl ester	822-23-1	0.92 ± 0.00 A	1.90 ± 0.02 B	0.89 ± 0.04 A	2.12 ± 0.08 C	KI	2218	2211	
43.25	N,N-Dimethylpalmitamide	3886-91-7	1.83 ± 0.16 A	2.80 ± 0.24 BC	1.45 ± 0.19 A	3.30 ± 0.25 BC	MS			87, 55, 43, 72
43.57	Cyclononasiloxane, octadecamethyl-	556-71-8	0.71 ± 0.12 A	0.64 ± 0.78 A	0.67 ± 0.09 A	0.95 ± 0.57 A	MS			57, 43, 69, 81, 147, 429, 355, 281

43.86	Heptacosane, 1-chloro-	62016-79-9	-	1.71 ± 1.57	-	-	MS	57, 43, 71, 85, 97, 29
45.15	Octadecane, 3-ethyl-5-(2-ethylbutyl)-	55282-12-7	-	-	0.12 ± 0.00 A	1.13 ± 0.72 B	MS*	57, 71, 43, 85, 97, 123, 183
45.46	Methyl dehydroabietate	1235-74-1	-	-	0.28 ± 0.00	-	MS	239, 57, 43, 69
46.89	Tetracosane	646-31-1	6.61 ± 1.00 A	8.53 ± 0.21 BCD	7.23 ± 0.61 ADE	8.74 ± 1.03 BCE	MS	57, 71, 43, 85, 97, 113, 127, 210
47.24	2-Bromooctadecanal	56599-95-2	0.34 ± 0.01	-	-	-	MS	57, 43, 69, 210
47.08	17-Pentatriacontene	6971-40-0	0.59	1.62 ± 0.05 A	0.80 ± 0.13 B	-	MS	43, 55, 69, 83, 97, 111, 125
47.41	Z-(13,14-Epoxy) tetradec-11-en-1-ol acetate	863489-15-0	-	-	-	1.94 ± 0.16	MS	177, 161, 149, 340, 121, 57, 41, 97, 69
49.53	Tetratetracontane	7098-22-8	1.04 ± 0.26 A	1.57 ± 0.08 A	1.26 ± 0.17 A	1.91 ± 0.35 A	MS	57, 71, 43, 85, 97, 111, 127
50.17	Cyclononasiloxane, octadecamethyl-	556-71-8	0.79 ± 0.51 A	1.08 ± 0.03 A	0.63 ± 0.06 A	0.80 ± 0.11 A	MS	57, 43, 69, 81, 147, 429, 355
50.75	Diisooctyl phthalate	131-20-4	0.2	1.51 ± 0.11 A	0.49 ± 0.00 B	1.77 ± 0.26 C	MS	149, 57, 43, 71, 83, 95, 167
50.95	Hexanedioic acid, dioctyl ester	123-79-5	0.63 ± 0.05 A	-	0.55	1.53 ± 0.27 B	MS	129, 55, 43, 71, 111, 241
51.23	1,54-Dibromotetrapentacontane	852228-22-9	0.54 ± 0.26	-	0.34	-	MS	57, 43, 69, 71, 85, 95
52.03	Hentriacontane	630-04-6	7.04 ± 1.75 A	8.33 ± 0.38 A	7.46 ± 0.32 A	8.39 ± 0.48 A	MS	57, 43, 71, 85, 97, 111, 125, 207
53.16	Cyclononasiloxane, octadecamethyl-	556-71-8	0.59 ± 0.09 A	0.57 ± 0.38 A	0.47 ± 0.02 A	0.80 ± 0.36 A	MS	57, 43, 69, 81, 147, 429, 355
54.43	Nonacosane	630-03-5	-	1.90 ± 0.97 A	1.30 ± 0.11 A	2.59 ± 0.32 A	MS	57, 71, 43, 85, 97
54.75	Phthalic acid, nonyl oct-3-yl ester	20548-62-3	0.43 ± 0.09 A	0.81 ± 0.03 BC	0.18	0.82 ± 0.03 BC	MS	149, 57, 55, 43
55.85	1,3-Benzenedicarboxylic acid, bis(2-ethylhexyl) ester	137-89-3	1.40 ± 0.26 A	3.36 ± 0.27 BC	1.55 ± 0.11 A	2.94 ± 0.47 BC	MS	149, 167, 57, 71
60.85	Hexatriacontane	630-06-8	7.99 ± 1.61 A	7.92 ± 0.00 A	8.53 ± 0.06 A	-	MS	43, 57, 71, 85, 97, 113, 127, 141, 155, 207, 281
65.54	Tetratriacontane	14167-59-0	6.47 ± 2.45 A	5.58 ± 0.76 A	7.18 ± 0.63 A	0.69 ± 0.36 A	MS	57, 71, 43, 81, 97, 111, 127, 141, 253
71.18	Tetratetracontane	7098-22-8	4.51	2.05 ± 2.63 A	-	-	MS	57, 71, 85, 43, 97, 111, 207, 125
71.65	Hexatriacontane	630-06-8	6.03	2.72 ± 0.00 A	5.27 ± 0.48 B	5.53 ± 0.55 B	MS	57, 71, 43, 85, 97, 11, 207, 281
74.10	Sesquiterpene lactone (epoxidized)*	-	1.93 ± 0.63 A	0.91 ± 0.86 A	2.05 ± 0.22 A	3.44 ± 1.02 B	MS	Tentatively identified based on MS fragmentation 385, 69, 83, 97
	**Total identified (%)**		74.51	85.81	71.89	83.07		

**Alkanes (%)**	52.32	59.45	50.64	35.76
**Esters (%)**	12.7	13.73	8.06	20.66
**Phenols (%)**	4.8	6.31	6.6	16.78

PLE (pressurized liquid extraction), MAE (microwave-assisted extraction), DME (dynamic maceration extraction), UAE (ultrasound-assisted extraction), IC (Identification Criteria), KI (Kovats Index) and MS (Mass Spectra); MS*(compound identified based on mass spectra from the reference [109] and [110]. * Retention indices were not determined due to the absence of alkane standards
