# Supplementary material for: Chemical Composition, Antioxidant and Antimicrobial Activity of Piper carpunya and Simira ecuadorensis: A Comparative Study of Four Extraction Methods
Source: Plants (Basel). 2025 Aug 14;14(16):2526. doi: 10.3390/plants14162526 (PMC12389035; doi:10.3390/plants14162526)
Supplement: Supplementary file 1 [file plants-14-02526-s001.zip › Tables S3 and S4.pdf]

Table S3. Summary of previous studies on *Simira ecuadorensis* and related species.

Species	Part used	Type of extract	Analysis method	Observed activity	Reference
Simira ecuadorensis	Leaves	Etanolic	Folin-Ciocalteu colorimetric method	Total Phenolic Content=346.28 mg GAE/100 g dry weight	[29]
			Disc diffusion assay	Inhibition of *Vibrio parahaemolyticus*	
Simira ecuadorensis	Leaves	Etanolic<br>Ethanol-water<br>Aqueous extracts	Agar diffusion method	Inhibition of: *Listeria mesenteroides*, *Shewanella* sp., *Yersinia enterocolitica*, *Clostridium perfringens*, and *Bacillus cereus*	[14]
			Challenge Test	The ethanol-water was effective against *Shewanella putrefasciens* in fish products (burgers and broth). The aqueous extract (spray-dried) was effective against *Campylobacter jejuni* in chicken broth.	
Simira sampaioana	Wood	Hexane<br>MeOH	Column chromatography<br>gas chromatography-mass spectrometry (GC-MS)	Phytochemical study isolate: Angelocunhol, 11β,12α-dihydroxy-2,4(18),15-erythroxylatrien-1-one, simiran B, harman, maxonin, isomalindine, malindine, sitost-4-en-6-ol-3-one, stigmat-4,22-dien-6-ol-3-one, campest-4-en-6-ol-3-one, sitost-4-en-3-one, stigmast-4,22-dien-3-one, campest-4-en-3-one, β-sitosterol, stigmasterol and stigmast-4,22-dien-3-ol	[25]
Simira grazielae	Wood	MeOH	NMR<br>Column chromatography<br>gas chromatography-mass spectrometry (GC-MS)	β-sitosterol, estigmasterol, 3-O-β -D- glucopiranosil sitosterol, siringaldehído, 3,4,5-trimetoxifenol, 6-O-vanilloil tacósido , siringaresinol, isofraxidina, escopoletina, umbeliferona, 7-hidroxi-8-metoxicumarina, 5,7-dimetoxicumarina, harman, ofiorina B y N-acetilserotonina.	[111]
Simira eliezeriana	Bark	MeOH	NMR, Column chromatography, GC-MS	Simirane A, simirane B, sitosterol, stigmasterol, campesterol,coniferaldehyde, vanillin, pinoresinol and harman	[112]

Table S4. Summary of previous studies on *Piper carpunya* and related species.

Species	Part used	Type of extract	Analysis method	Observed activity	Reference
Piper lenticellosum	Leaves and spikes	Essential oil	GC/MS<br>Disk diffusion agar method	Constituents: piperitone, 1,8 cineole, limonene, safrole and α-pinene.<br>Activity against Staphylococcus aureus, Escherichia coli and Klebsiella pneumoniae	[18]
Piper carpunya	Leaves and spikes	Essential oil	GC/MS	major constituents: α-terpinene, p-cymene, 1,8-cineole, safrole and spatulenol	[30]
Piper carpunya	Leaves	Essential oil	GC/MS<br>DPPH<br>Broth microdilution method<br>Ames test	Main constituents: piperitone, limonene, elemicin, and β-phellandrene.<br>DPPH antioxidant assay showed weak radical scavenging ability (IC50)<br>Low bioactivity against yeast (*Candida albicans,Candida glabrata*, and *Candida albicans*)<br>Ames test: evidenced the safety of the EO concerning genotoxicity.	[19]
Piper carpunya	Leaves	Essential oil	GC/MS<br>DPPH, ABTS<br>Broth microdilution method	The main constituent of the essential oil was 1,8-cineole<br>strong activity against *Klebsiella pneumoniae.*<br>Absence of antioxidant activity(DPPH, ABTS)	[68]
Piper carpunya	Leaves	Etanolic	Agar diffusion method	Activity against: *Staphylococcus aureus*, *Escherichia coli* and *Pseudomona aeruginosa*, and *Candida albicans*	[24]
Piper xylosteoides	Leaves	Essential oil	Broth microdilution method<br>GC/MS	Eleven compounds were identified in the essential oil, with safrole and α-pinene being the main components.<br>Weak antibacterial activity against: *Bacillus cereus*, *Staphylococcusaureus*, *Escherichia coli* and *P. aeruginosa*	[113]
Piper ecuadorense	Leaves	MeOH	broth<br>microdilution method	Strong anti-fungal activity against *Trichophyton mentagrophytes* and *Trichophyton rubrum*	[114]
Piper marginatum Jacq	Leaves and inflorescenc es	Etanolic	Agar diffusion method	Antimicrobial Activity against: *Porphyromonas gingivalis*, *Fusobacterium nucleatum*, and *Prevotella intermedia*	[115]
