# Supplementary figures and images for: Chemical Composition, Antioxidant and Antimicrobial Activity of Piper carpunya and Simira ecuadorensis: A Comparative Study of Four Extraction Methods
Source: Plants (Basel). 2025 Aug 14;14(16):2526. doi: 10.3390/plants14162526 (PMC12389035; doi:10.3390/plants14162526)

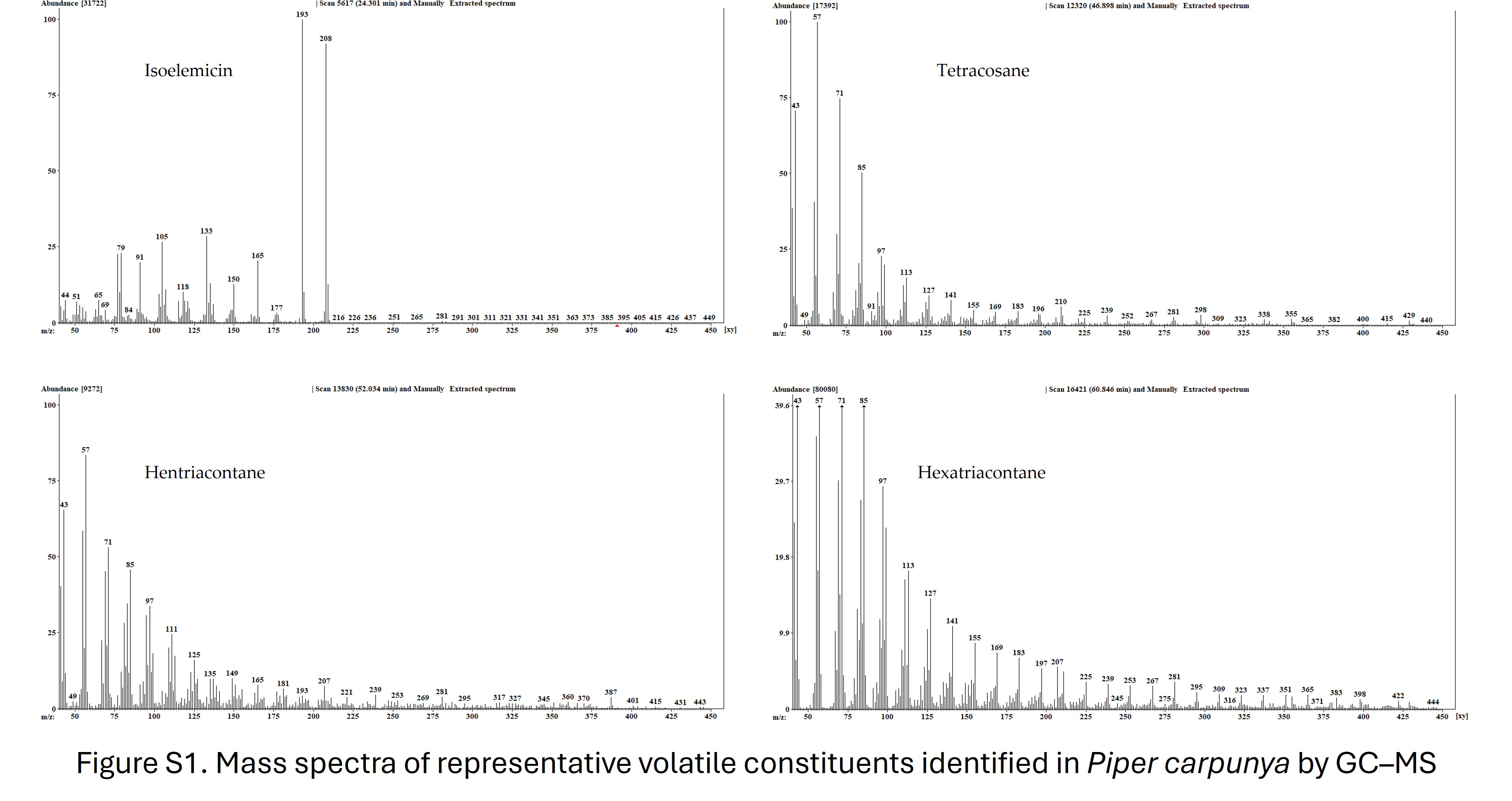

Supplement: Supplementary file 1 [file plants-14-02526-s001.zip › Figure S1.jpg]

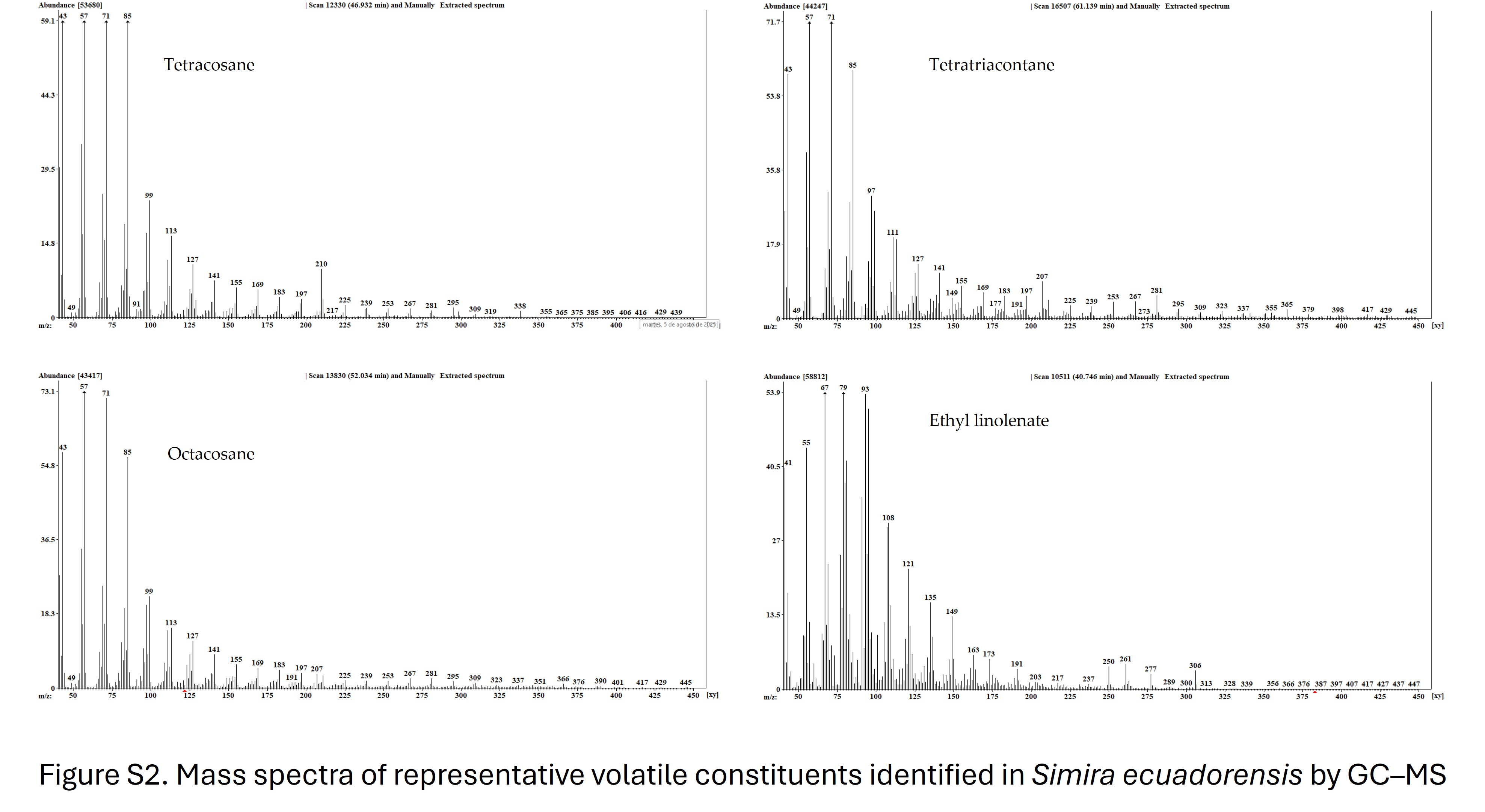

Supplement: Supplementary file 1 [file plants-14-02526-s001.zip › Figure S2.jpg]

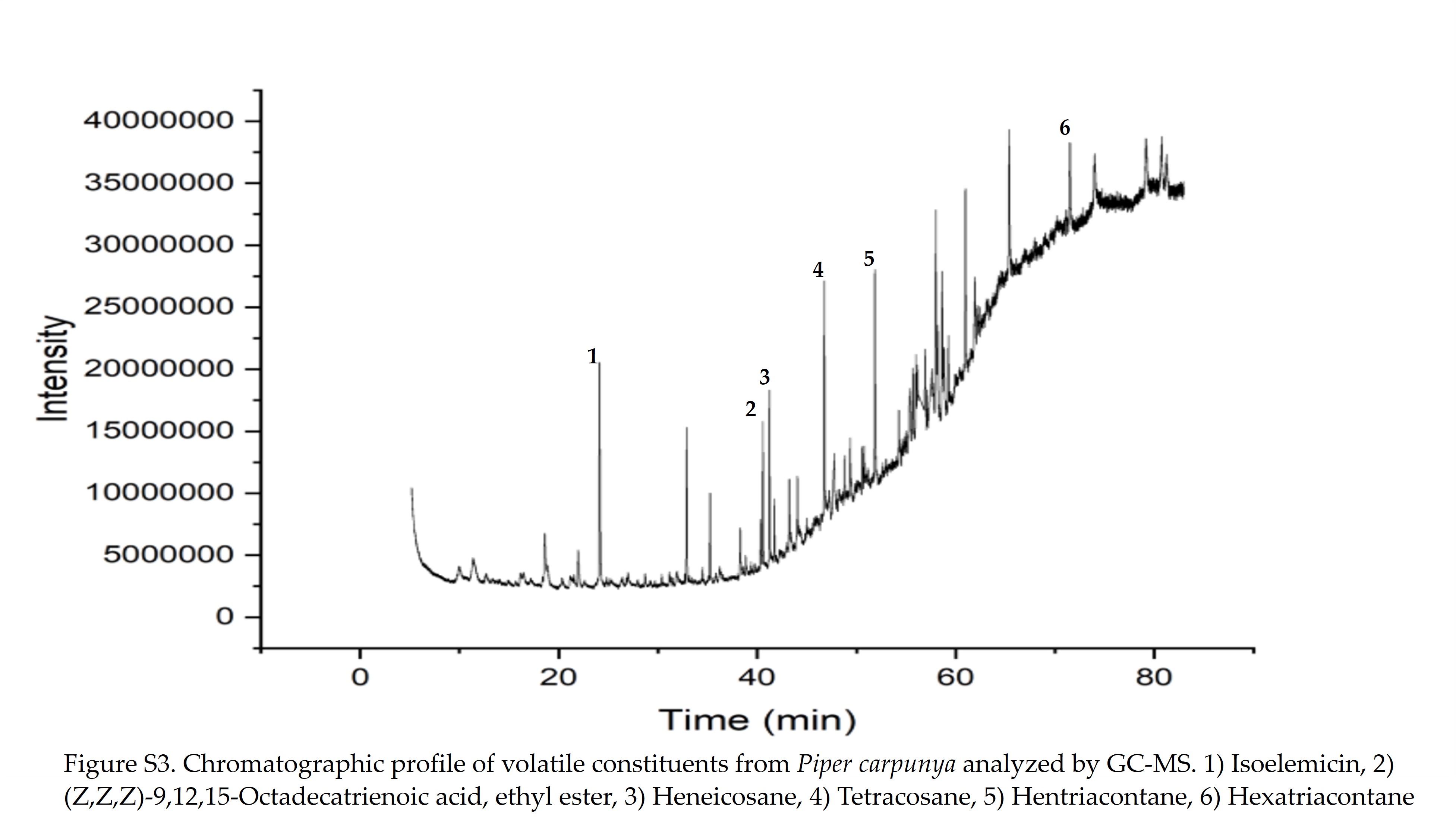

Supplement: Supplementary file 1 [file plants-14-02526-s001.zip › Figure S3.jpg]

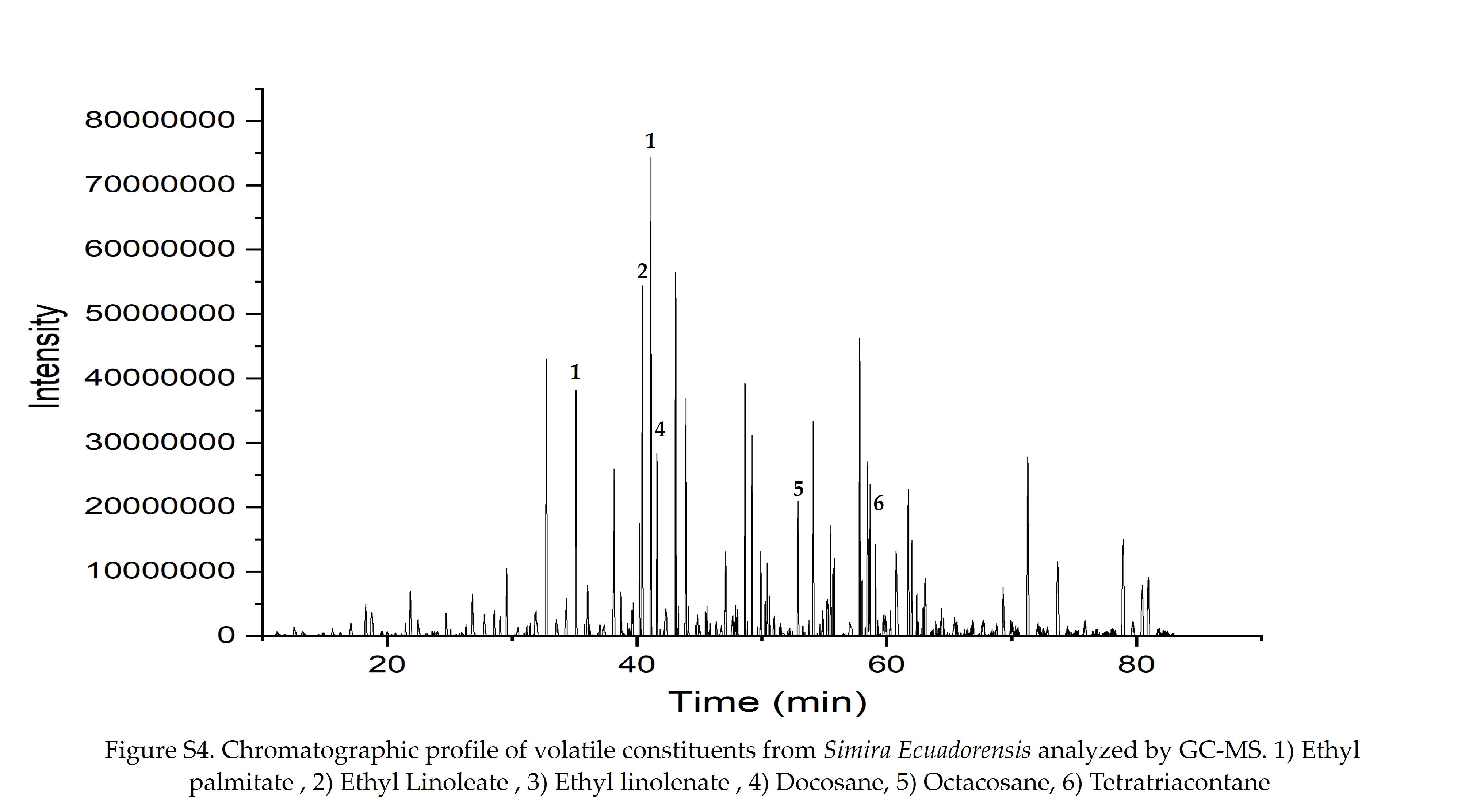

Supplement: Supplementary file 1 [file plants-14-02526-s001.zip › Figure S4.jpg]
